# Possible Use of Phytochemicals for Recovery from COVID-19-Induced Anosmia and Ageusia

**DOI:** 10.3390/ijms22168912

**Published:** 2021-08-18

**Authors:** Sachiko Koyama, Kenji Kondo, Rumi Ueha, Hideki Kashiwadani, Thomas Heinbockel

**Affiliations:** 1 Department of Chemistry, Indiana University, Bloomington, IN 47405, USA; 2 Department of Otolaryngology, Faculty of Medicine, The University of Tokyo, Tokyo 113-8655, Japan; uehar-oto@h.u-tokyo.ac.jp; 3 Swallowing Center, The University of Tokyo Hospital, Tokyo 113-8655, Japan; 4 Department of Physiology, Graduate School of Medical and Dental Sciences, Kagoshima University, Kagoshima 890-8544, Japan; danny@m3.kufm.kagoshima-u.ac.jp; 5 Department of Anatomy, College of Medicine, Howard University, Washington, DC 20059, USA

**Keywords:** COVID-19, anosmia, ageusia, smell training, taste training, phytochemicals, essential oils, diets, anti-inflammatory effects, anti-viral effects

## Abstract

The year 2020 became the year of the outbreak of coronavirus, SARS-CoV-2, which escalated into a worldwide pandemic and continued into 2021. One of the unique symptoms of the SARS-CoV-2 disease, COVID-19, is the loss of chemical senses, i.e., smell and taste. Smell training is one of the methods used in facilitating recovery of the olfactory sense, and it uses essential oils of lemon, rose, clove, and eucalyptus. These essential oils were not selected based on their chemical constituents. Although scientific studies have shown that they improve recovery, there may be better combinations for facilitating recovery. Many phytochemicals have bioactive properties with anti-inflammatory and anti-viral effects. In this review, we describe the chemical compounds with anti- inflammatory and anti-viral effects, and we list the plants that contain these chemical compounds. We expand the review from terpenes to the less volatile flavonoids in order to propose a combination of essential oils and diets that can be used to develop a new taste training method, as there has been no taste training so far. Finally, we discuss the possible use of these in clinical settings.

## 1. Introduction

The outbreak of the coronavirus, SARS-CoV-2, has escalated into a worldwide pandemic. Over 173 million people in the world have contracted the virus, causing over 3.73 million deaths as of 7 June 2021. In December 2019, when the first reports on the outbreak came out, the symptoms reported were shortness of breath, cough, fever, and diarrhea. However, it has become clear that many patients who contract the virus are asymptomatic and yet contagious [1,2]. In addition, the symptoms of the symptomatic patients were found to be more varied than when first reported [3]. Recent studies have shown that this could be due to the spike mutation protein D614G and B.1.1.7 (now called the alpha variant, first outbreak in the U.K.) in SARS-CoV-2, which appears in a higher viral load in the upper respiratory tract compared to the lungs [4,5,6,7]. In less than one year, the SARS-CoV-2 coronavirus has mutated several times resulting in genetically different variants with distinct geographic separation. The variants are named by using letters of the Greek alphabet. The Beta variants (first outbreak in South Africa), the Gamma variants (first outbreak in Brazil), and now the Delta variants (first outbreak in India) are causing a new increase in the number of cases around the world.

Following these changes, the symptoms have also shown some changes. Among the various symptoms reported was the unique sudden and novel loss of the senses of smell (anosmia) and taste (ageusia) [1,8,9,10,11]. The percentage of patients showing anosmia or ageusia was especially high in the otherwise asymptomatic patients and the patients experiencing mild levels of other symptoms. Recent reports have shown that almost 45% of the coronavirus-induced disease (COVID-19) patients had only anosmia and ageusia as symptoms [2,12]. Moreover, a recent study has shown that 98% of COVID-19 patients showed some smell dysfunction [10] indicating that, following the outbreak of COVID-19, a large number of patients lost their olfactory sense or had malfunction in their olfactory sense. Recent reports on the variants also suggest variant-dependent differences in the COVID-19-induced chemosensory dysfunction. There is a strong clinical need to develop medication treatments to help the recovery of the chemical senses, smell and taste.

One of the treatment methods, which has been used for decades for patients with post-viral anosmia or hyposmia, is smell training [13,14,15]. So far, there have been no treatment methods comparable to smell training for ageusia that can be called “taste training”. Smell training uses essential oils, and traditionally the types of essential oils most often used have been those of rose, lemon, eucalyptus, and clove [13], although there are various other choices available to use. More recently, studies have revealed that many of the chemical constituents of essential oils have bioactive properties; for example, suppressing neuropathic pain and inflammation, anti-viral effects, anxiolytic effects, and enhancing regeneration by increased re-epithelialization of cutaneous wounds through cell proliferation and migration [16,17]. Recent studies have started to determine the mechanisms of action as well [18,19,20,21,22]. Studies on olfactory neuroscience and adult neurogenesis have made a dramatic development from the 1990s. Studies on how we sense taste also followed from the 2000s. If we can incorporate what we have learned during these more recent years with the study of natural products (botanical pharmacology or phytochemistry), we may be able to develop smell training and taste training that incorporates our knowledge of the neuroscience of olfaction and gustation and our knowledge of the bioactive properties of the chemical constituents of essential oils and plants. Besides, each person has their own history of olfactory experience, genetically inherited characteristics, and personally different symptoms and levels of symptom severity. If we can understand the bioactive properties of the chemical constituents, it may be possible to develop a new concept which can be called a “precision olfactory and taste training” (we coined the phrase “precision olfactory and taste training” following the concept of the “Precision Medicine Initiative”. The “Precision Medicine Initiative” started from the 2015 State of the Union address by President Obama of the U.S. (https://obamawhitehouse.archives.gov/precision-medicine (accessed on 8 January 2021)). According to the Precision Medicine Initiative, Precision Medicine is an approach for disease treatment and prevention that considers individual variability (https://obamawhitehouse.archives.gov/precision-medicine (accessed on 1 January 2021) and https://medlineplus.gov/genetics/understanding/precisionmedicine/definition/ (accessed on 1 January 2021)). In this review, we are considering that each symptom of anosmia and ageusia contains individual variability, hence the name “precision olfactory and gustatory training”), utilizing the knowledge of the chemical compounds, the symptoms of each person, and other factors related to their sensory dysfunction. In this review, we will summarize studies on the olfactory and gustatory system, COVID-19-induced anosmia and ageusia, and the bioactive chemical compounds in herbal plants and essential oils, focusing on terpenes and flavonoids. At the end, we propose a new smell and taste training based on the bioactive properties of these chemicals, which hopefully enhances the recovery of the senses of olfaction and gustation.

## 2. Post-Viral Anosmia

### 2.1. Diagnosis and Symptoms

#### 2.1.1. Symptoms

Post-viral olfactory dysfunction (PVOD) is defined as the persistence of olfactory disturbances after a viral infection of the upper respiratory tract, even after the symptoms of upper respiratory tract inflammation have disappeared [23,24]. Patients with acute upper respiratory tract infections are aware of rhinitis symptoms such as nasal obstruction, nasal discharge, sneezing, and often notice a loss of sense of smell as well. In most cases, olfactory loss is a result of an airflow problem in the olfactory cleft due to mucosal swelling and increased nasal discharge. The patient recovers with the disappearance of rhinitis symptoms. In a small number of patients, however, the olfactory disturbance persists even after the rhinitis symptoms have disappeared.

PVOD is one of the three most common causes of olfactory dysfunction in clinical settings, along with chronic rhinosinusitis and traumatic olfactory dysfunction [25,26,27]. It is more common in middle-aged and older women, with the average age of patients in their 50 s [25,26,27,28]. The reason for such a high incidence in women is currently unknown.

Patients often do not seek medical consultation immediately after noticing olfactory disturbance, thinking that the olfactory disturbance will eventually improve. Therefore, there is a time lag of several weeks or months between the onset of the upper respiratory tract infection and the visit to a clinic [23,24], and there is often no evidence of abnormality in the local endoscopic results for the nasal cavity or sinonasal imaging studies at the time of the visit. Therefore, the history of olfactory loss after upper respiratory tract infection is of primary importance for the correct diagnosis of this disease.

PVOD is often associated with qualitative olfactory dysfunction, including parosmia, which is a sensation where an odorant is perceived differently than it used to smell, and phantosmia, which is a sensation of some odor in the absence of any odorant source. For example, Reden et al. reported that in a total of 392 patients with olfactory impairment, parosmia was more frequent (56%) in PVOD than traumatic olfactory dysfunction (14%) and olfactory dysfunction due to rhinosinusitis (28%) [29]. Parosmia occurs more frequently than phantosmia [29,30,31]. Qualitative olfactory dysfunction may occur simultaneously with olfactory loss, or it may be delayed.

#### 2.1.2. Pathophysiology and Viruses

The exact pathophysiology of PVOD is not yet fully understood, but it is thought to be caused by viral insult on the olfactory neural tissue [23,24]. Based on histological examination of human olfactory mucosal biopsies and animal models, both the neuroepithelium of the olfactory mucosa and central olfactory pathway could be involved in the pathogenesis of PVOD [32,33]. Viral infection could damage the neural tissue directly, or induce an immune reaction of the host and cause secondary tissue damage by inflammatory cytokines and mediators [34,35,36].

As for the causative viruses, Suzuki et al. collected nasal secretions from patients with PVOD and analyzed them by polymerase chain reaction (PCR) assays. They detected rhinovirus, coronavirus, parainfluenza virus, and EB virus, with rhinovirus being most frequently detected [37]. Tian et al. examined the viruses in olfactory cleft mucus sample using a multiplex PCR kit, and detected rhinovirus most frequently [38]. Konstantinidis et al. reported that the incidence of PVOD has a seasonal fluctuation and the seasonal peak of PVOD appears to correlate with the peak of occurrence of influenza [39].

Sugiura et al. also examined the monthly incidences of PVOD and monthly incidences of virus isolation. The former was highest in June, and parainfluenza virus type 3 was more frequent during the same period [28]. Wang et al. also performed reverse transcription (RT)-PCR on the mucosa of the inferior turbinate and found that parainfluenza virus type 3 was detected in 88.0% of patients with PVOD, compared to only 9.1% of controls [40].

Influenza virus infection can be rapidly and clearly diagnosed using a detection kit. In a retrospective analysis of 587 PVOD cases in North America, Potter et al. divided the cases into influenza and non-influenza (NI) groups and examined the onset time. For influenza-related cases, both the prevalence and magnitude of smell dysfunction were highest in the colder months. On the other hand, for NI-PVOD-related cases, prevalence was higher in warmer months but the magnitude of dysfunction was higher in colder months [41].

The mechanism that causes SARS-CoV-2 to result in olfactory dysfunction more frequently than other upper respiratory viruses is not fully understood. It may be associated with the distribution of receptor molecules that are used for viral entry into cells, as discussed below in Section 2.3. It may be also related to the extent of viral infection and the host immune response. A recent study reported that the induction of antiviral innate immune molecules in the nasal epithelial cells at the early stages of infection was lower for SARS-CoV-2 than for influenza virus [42]. This suggests that viral elimination may be delayed and viral replication may proceed in the mucosa for a long time, which may be related to the development of olfactory disturbances characteristic of COVID-19.

#### 2.1.3. Diagnosis

As mentioned above, the diagnosis of PVOD only occurs when olfactory disturbances persist, even after the symptoms of upper respiratory tract infection have disappeared. Therefore, the role of clinical examinations is to rule out other causes of olfactory disturbances and to assess the severity of olfactory dysfunction. Examinations for the former role include nasal endoscopy to confirm the absence/presence of a lesion in the nasal cavity, especially in the olfactory cleft, and a computerized tomography (CT) scan of the paranasal sinuses. Both of them often show no abnormality in the patients with PVOD. For the latter purpose, olfactory tests are used to evaluate olfactory threshold, discrimination and identification ability. Various types of olfactory tests are used across the world, since the type of the odors familiar to the population is different among the different cultural backgrounds. In the United States, the University of Pennsylvania Smell Identification Test (UPSIT) is often used, while in Europe, the Sniffin’ Sticks test is used. In Japan, T&T olfactometry is the standard olfactory test battery. It is difficult to directly compare the data obtained from each test due to the differences in testing methods and criteria for determining the degree of impairment. In general, the degree of smell loss in PVOD is often milder than that of traumatic olfactory dysfunction or chronic sinusitis [25,26,27], and the severity of olfactory impairment does not necessarily correlate with the degree of subjective impairment. Patients with a moderate impairment on olfactory examination may complain of little or no sense of smell.

### 2.2. Upper Respiratory Tract as a Major Gate for SARS-CoV-2

**Figure 1:**
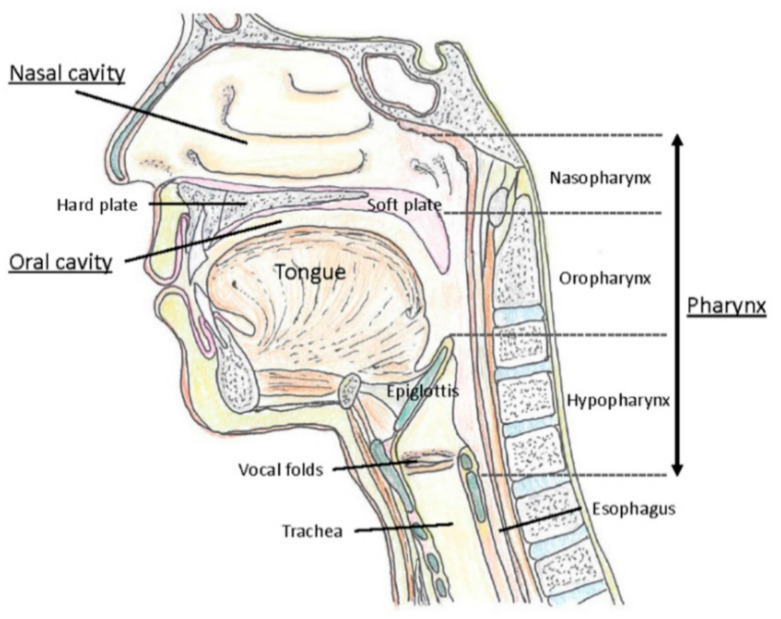
The upper respiratory tract.

The upper respiratory tract refers to the nasal cavities, sinuses, pharynx including tonsils, and larynx (Figure 1). In contrast, the lower respiratory tract consists of the trachea and the lungs with their substructures including bronchi, bronchioles, and alveoli. The nasopharynx is mainly lined by ciliated columnar epithelium but stratified squamous epithelium occurs at its lower end where it joins the oropharynx. The oropharynx and hypopharynx are lined by largely non-keratinized stratified squamous epithelium. The lateral walls of the oropharynx are composed of the palatine tonsils and tonsillar pillars [43]. The laryngeal epithelium corresponding to the mechanically exposed areas, including the upper part of the epiglottis and the vocal cords, consists of stratified squamous nonkeratinized epithelium. In the rest of the larynx, including the lower part of the epiglottis, the laryngeal ventricle, and infraglottic areas, the epithelium is ciliated columnar pseudostratified with goblet cells [44].

Viral loads of SARS-CoV-2 have been found to be high in the upper respiratory tract, especially in the nose and nasopharynx, whereas that of SARS-CoV, which emerged in Guangdong Province, China, in 2002, was reported to be high in the lower respiratory tract [45]. As with the nasal cavity, in general, the pharynx and larynx are thought to be the sites where the virus can easily bind. Upper respiratory tract viral load could identify high-risk patients with COVID-19, as high viral load in the upper respiratory tract is associated with severe disease [46]. However, it has been reported that viral load levels in the upper airway do not differ between the patients with mild levels of COVID-19 symptoms and those with severe/critical levels of them [47], thus there is still hesitation to use the levels of viral load as a prognostic marker of COVID-19. SARS-CoV-2 viral load in the upper respiratory tract appeared to peak in the first week of illness, whereas that of SARS-CoV and MERS-CoV peaked at days 10–14 and 7–10, respectively [48]. SARS-CoV-2 shedding duration was positively associated with age [48,49]. Interestingly, no study detected live virus beyond day 9 of illness despite persistently high viral loads, which was inferred from the rapid approach to the threshold level, i.e., the cycle threshold values, with fewer numbers of amplification cycles [48]. Duration of viral genetic shedding was shorter from the upper respiratory tract specimens (9–20 days) than those in the lower respiratory tract (14–34 days) [48,49].

Angiotensin-converting enzyme 2 (ACE2) is a receptor, which is responsible for the cellular entry of SARS-CoV-2, and transmembrane protease serine 2 (TMPRSS2) is a protease, which facilitates viral entry into the host cells. In the upper respiratory tract, ACE2 and TMPRSS2 are expressed in many tissues with various degrees of expression. The oral mucosa including the palate displays mild to moderate ACE2/TMPRSS2 expressions in the epithelium [50,51]. The tonsil expresses weakly ACE2 and strongly TMPRRS2 in the epithelium [50,52]. In the epithelium of the hypopharynx, ACE2 and TMPRSS2 are mildly expressed in the superficial layer [50,51]. In the larynx, the epithelial lining, laryngeal glands, and lamina propria express ACE2 [52,53]. Especially in the epithelium of the glottis, there are rather few ACE2-positive cells, whereas TMPRSS2-positive cells are in more abundance [50,51,53].

The common pharyngo-laryngological manifestations in COVID-19 patients are pharyngodynia (10–12%), pharyngeal erythema, tonsil enlargement, and dysphonia [8,54,55,56]. Except for pharyngodynia, the incidence of each symptom is relatively low. Regarding dysphonia, females tend to develop dysphonia more frequently than males, and smoking is associated with dysphonia in COVID-19 [8]. It must be taken into account that both pulmonary and laryngological involvements in patients with COVID-19 can affect speech function [56]. The expression of ACE2/TMPRSS2 in the mucosa of the pharynx and larynx may explain the involvement of mild oral and throat symptoms in patients with COVID-19.

### 2.3. COVID-19-Induced Anosmia

There are several possibilities for the causation of COVID-19-induced anosmia. One is damage to the morphology of the olfactory epithelium, where the olfactory sensory neurons reside. The second possibility is damage to the morphology of the olfactory bulb, which will obstruct signal transfer to the brain. Furthermore, the third possibility is the inflammatory immune response, which can weaken the olfactory system.

Damage to the olfactory epithelium can be caused by direct infection of olfactory sensory neurons, infection of the surrounding sustentacular cells causing damage to the morphology of these cells which eventually will cause damage to the olfactory sensory neurons, and the inflammatory cytokines causing a malfunction of olfactory sensory neurons [57].

For the entry of the SARS-CoV-2 virus into the host cells, it is now well known that the spike (S) glycoprotein of SARS-CoV-2 virus binds to ACE2, a metalloproteinase ectoenzyme that regulates angiotensin II, which allows the virus to enter the host cells through endocytosis [58,59]. Serine protease TMPRSS2 and proprotein convertase furin also have key roles in priming the S glycoprotein, which is required for host cell entry [59,60]. In the olfactory epithelium, ACE2 is expressed in the sustentacular cells but not in the olfactory sensory neurons [61,62]. There are also studies that have found sparse expression of ACE2 in the olfactory sensory neurons but not as profoundly as in the supporting cells [63]. ACE2 and TMPRSS2 were most intensely expressed in the supporting cells and in the Bowman’s glands [63,64,65]. Furin was also found expressed greatly in the supporting cells and in the Bowman’s glands [63].

This distribution of the cellular expression of ACE2 suggests that the malfunction of the olfactory sensory neurons is due to damage to their morphology from virus infection of the supporting cells [66] and/or the inflammatory cytokines [67]. Proinflammatory cytokine levels measured using enzyme-linked immunosorbent assay (ELISA) in olfactory epithelium samples from patients deceased due to COVID-19 were significantly higher than the control group patients whose samples were collected by biopsy during routine nasal surgeries [67], which supports this hypothesis. Studies using hamsters as an animal model have shown that, although ACE2 is expressed in the supporting cells and not in the olfactory sensory neurons, hamsters that were inoculated with virus had completely lost the cilia of the olfactory sensory neurons and particles of virus were found attached to or shedding off from the bare surface of these cells [68]. It could be that, at an early stage, the symptom of anosmia was caused by the inflammation, and then the infection proceeded and the replication of the virus increased, causing extensive expansion of the infected area and extensive morphological damage that caused loss of the cilia from the olfactory sensory neurons. Although some patients recover their senses within about 2 weeks, many patients suffer loss or malfunction of their senses for long term [3]. This damage that requires regeneration could be the reason for the long-term malfunctioning in the senses. Studies using brain organoids show that the neuronal death did not colocalize with virus infection [69]. The pathways related to hypoxia were up-regulated in the non-infected cells around SARS-CoV-2 infected cells whereas SARS-CoV-2 infected cells showed up-regulation in the pathways related to hyperoxia, indicating their hypermetabolic state [69]. Possibly, the viral infection and the replication of the virus in the host cells of the olfactory epithelium cause “locally hypoxic regions, which aids in lowering the threshold for tissue damage in the context of an already oxygen-deprived state”, such as the brain organoids [69]. Metabolic alteration following viral infection has been known for decades [70,71]. Although there are some differences due to the species of virus [70,72,73,74], an increase in glycolysis is common to many types of viruses [70]. A recent study has shown using kidney epithelial cells and lung air-liquid interface cell models that infection by SARS-CoV-2 increased the pyruvate carboxylase expression, stimulated the tricarboxylic acid (TCA) cycle, and enhanced the mechanistic target of rapamycin complex 1 (mTORC1) activity [75]. Changes in the metabolic pathways induce elevated intracellular levels of reactive oxygen species (ROS), i.e., oxidative stress, leading to damages to lipids, proteins and DNA. This suggests that metabolic alteration can take place and negatively affect the cells surrounding the infected cells. This is not only the case for brain organoids, but in various parts of the body, including the nasal cavity, where SARS-CoV-2 infection takes place. Thus, the inflammation and the morphological damage, first in the supporting cells and then the olfactory sensory neurons possibly through hypoxia are causing the COVID-19-induced anosmia. The larger the damage is, the longer it may take to regain the functions of the senses.

Following the outbreak of SARS-CoV-2, chemosensory loss has been well documented. Whether this is because of the large difference in the infectiousness between SARS-CoV-2 and previous human coronavirus, or because there are some mechanistic differences that cause higher chemosensory dysfunction, are not known. The SARS-CoV-2 spike glycoprotein is 76% homologous to those of SARS-CoV [76]. The SARS-CoV-2 is far more infectious than SARS-CoV and the variants of the SARS-CoV-2 are also more contagious than the original SARS-CoV-2 [77]. The mutation in the RBD of the S-glycoprotein of the virus causing differences in the binding affinity to ACE2 [78,79] could be one of the reasons for this increased contagiousness.

There are also factors on the host side. ACE2 is now well known as the receptor for both SARS-CoV and SARS-CoV-2 and for the variants of SARS-CoV-2 to enter host cells. There are variants of ACE2 which can cause differences in the binding affinity with the receptor binding domain (RBD) of the S-glycoprotein of the virus [80]. It is well known that mice cannot be used as model animals for SARS-CoV-2 transfection studies unless transgenic mice which express human ACE2 are used because of the low infection rate in mice. This suggests that species comparison of the genes that comprise ACE2 might provide us with important information on the binding affinity between ACE2 and the RBD of the S-glycoprotein of SARS-CoV-2, and thus the cell entry. Interestingly, and importantly, in a study which compared the binding of SARS-CoV-2 S-glycoprotein with ACE2 orthologs of various species expressed in A549 cells, it was found that the percentage of the gene shared with human ACE2 did not correlate with the infection rate in the animal species [81]. Instead, they found that there are key regions that affect the binding affinity, i.e., the hydroxyl group of Tyr (Y) at human ACE2 position 41 (H41Y) and the side-chain nitrogen atom of Q42 of human ACE2 (E42Q) were found to have critical roles in strengthening the binding with the RBD of the S-glycoprotein of SARS-CoV-2 [81]. Such species comparison may suggest genetic differences among individuals that affect the contraction of the virus or the severity of the symptoms.

Multiple other factors on the host side are known to affect the infection and replication of SARS-CoV-2 [82]. Genes involved in, for example, cholesterol homeostasis, were found to be important for the virus to enter the host cells efficiently [82], suggesting that the differences in the expression of these genes would affect the infection and severity of the symptoms of those who contracted the virus. There are possible roles of other proteins/peptides as the entry sites. Neuropilin-1 (NRP1) is expressed in abundance in the olfactory epithelium, binds to furin-cleaved substrates, and enhances infection by SARS-CoV-2 [83]. It is expressed more in the infected epithelial cells of COVID-19 patients than controls, and it is thought that NRP1 potentiates the attachment of the virus and enhances virus entry through ACE2 [83,84]. Integrin is a transmembrane receptor [85,86,87,88] and it is known to control uptake of extracellular vesicles and viruses [88]. The S glycoprotein of SARS-CoV-2 possesses the integrin-binding RGD (Arg-Gly-Asp) tripeptide motif, which is known for its roles in virus infection [85,89]. In addition, both ACE2 and integrins possess the short linear motifs that may enhance the internalization of the virus. Other than the endocytosis pathway, there is also a possibility that an autophagy process is involved in virus infection [90]. In case of integrin, studies have found that the phosphorylation of Ser^778^ located upstream of the hydrophobic motif strengthened binding to the autophagy-related protein 8 and the phosphorylation of Tyr^785^ located down-stream of the hydrophobic motif enhanced the affinity as well [86]. Sialic acid [76,84,91,92] is also known to serve as a binding site for the virus and there is also a concern for its possible involvement in the cytokine storm [93]. SARS-CoV-2 has the receptor binding domain S1A that binds to sialic acid (Neu5Ac). S1A binding to sialic acid is considered to facilitate cell entry most likely by tethering the virus on the host cell surface, helping in viral surfing [92,94]. The sialic acid linked to galactose by α-2,3 linkage (SAα-2,3) or α-2,6 (SAα-2,3) linkage is expressed in abundance in the lung and bronchus [95,96]. The SAα-2,6 is mostly expressed in non-alveolar cells whereas SAα-2,3 is expressed more in the alveolar cells [97]. These differences in the distribution and the difference in the binding affinity with different viruses are known to determine where the infection happens [95,97].

There is also evidence showing an interaction between the receptor binding domains of the S glycoprotein of the virus and CD147 [84]. These studies show that, although ACE2 is well known as the receptor for the SARS-CoV-2 virus, there are possibilities of various host cell entry sites and sites where binding supports attachment to host cells. These various binding sites are not expressed in limited locations but are rather ubiquitous, which could be one of the reasons for the high infectiousness and the high occurrence rate of anosmia and ageusia symptoms.

## 3. Perception of Odors

### 3.1. Perceiving Odors

#### Olfactory Neuroscience

**Figure 2:**
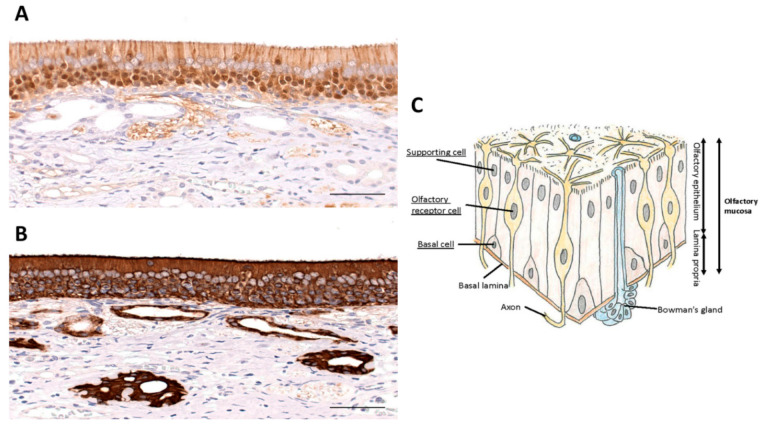
Human olfactory epithelium. (**A**) IHC staining with PGP9.5 showing neurons at the olfactory epithelium, which shows the olfactory sensory neurons. (**B**) IHC staining of cytokeratin18 showing the epithelial cells at the olfactory epithelium, showing supporting cells and Bowman’s glands. (**C**) Structure of olfactory epithelium showing the olfactory sensory neurons, supporting cells, basal cells and Bowman’s gland. Scale bars in (**A**,**B**) indicate 50 µm.

In order to understand the possible use of terpenes and flavonoids in the recovery from COVID-19-induced chemosensory dysfunction, it is important to briefly review the olfactory pathway. This pathway starts in the nose where the nostrils or nares are separated by a septum. The vestibule is the most anterior part of the nasal cavity which is enclosed by elastic cartilage and lined by a stratified squamous, keratinized epithelium. Further back, the nasal cavity is lined by respiratory epithelium, which is a pseudostratified, ciliated, columnar epithelium. The same type of epithelium is found further down the airways including the trachea and bronchi. Deep in the nasal cavity, our organ of smell is formed as a specialized epithelium, the olfactory epithelium (Figure 2), which sits on the superior conchae and presents as the olfactory area. Each nasal cavity has its own olfactory area in the roof of the nose. The olfactory epithelium is also a pseudostratified ciliated columnar epithelium. It houses olfactory sensory neurons, supporting cells (sustentacular cells), and basal stem cells.

Olfactory sensory neurons are bipolar neurons that bind and detect odorant molecules [98]. The axons of these neurons coalesce to form the olfactory nerve, cranial nerve I, that traverses the cribriform plate of the ethmoid bone, and projects to the ipsilateral olfactory bulb where the axons synapse on central neurons. Olfactory sensory neurons are surrounded by supporting or sustentacular cells. Olfactory sensory neurons are equipped with radiating cilia that emanate from their dendrites. In contrast, sustentacular cells have microvilli at their apical surface. The basal cells are found in the lower part of the epithelium and serve as precursor cells that actively divide to replace olfactory sensory neurons. This continuous replacement is needed because of the short life span of olfactory sensory neurons of 30–60 days [99]. Bowman’s glands are found in the connective tissue (lamina propria) underlying the olfactory epithelium. They send their ducts to the surface of the epithelium and secrete a serous fluid that immerses the cilia of olfactory sensory neurons in a mucus layer to trap odorant molecules and to prevent constant olfactory stimulation. Their secretion produces a fluid environment around the olfactory cilia to clear the cilia which facilitates the access of new odor substances. Furthermore, the mucus creates the ionic milieu around the cilia with odorant-binding proteins that trap odorants and bring them to the cilia.

Olfactory receptors need to be exposed to the external environment to detect evaporated chemicals. The peripheral olfactory organ is, therefore, always at risk of being injured by extrinsic pathogens and chemicals. On the other hand, olfaction plays an indispensable role in survival, contributing to food detection, predator avoidance, and mating in animals. To meet these diverse needs, the mammalian olfactory neural system has a unique regenerative capacity. The most distinct feature of this regenerative capacity is the continuous proliferation of basal cells in the neuroepithelium. Basal cells are a type of neural stem cell, which continuously undergo cell division even in undamaged conditions and give rise to new olfactory sensory neurons. When the neuroepithelium is injured, such proliferative activity is upregulated so the neuroepithelium is regenerated rapidly. In rats and mice the olfactory neuroepithelium morphologically recovers from experimentally-induced mucosal injury in about one month [100].

In spite of such a regenerative capacity, neural olfactory dysfunction in humans often lasts for months to years, and is sometimes permanent. The reason for such discrepancy is not clear, but the following possibilities may be associated: (1) it may take a longer time for the human olfactory neuroepithelium to recover from damage; (2) it may take time for the regeneration of central olfactory pathways following peripheral olfactory nerve regeneration, such as synaptic remodeling of olfactory nerves and mitral/tufted cells in the olfactory bulb, or circuit regeneration of inhibitory neurons.

Furthermore, the neurogenic potential of basal cells is affected by a variety of pathologic factors, including age-related changes, infection, and airway inflammation. For example, it has been reported that the number of Sox2-positive globose basal cells decreases in a mouse model of RS virus infection [101]. As such, there are various factors that could be involved in the persistent PVOD after viral clearance.

**Figure 3:**
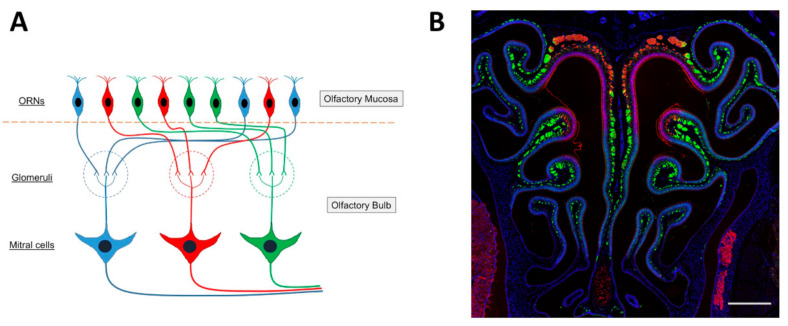
(**A**) Each olfactory sensory neuron expresses one type of olfactory receptor. The axons from olfactory sensory neurons with the same type of olfactory receptors extend to the olfactory bulb and form one glomerulus. The signaling is transferred to the brain from the glomerulus through mitral cells. (**B**) Immunofluorescence staining of coronal sections of a mouse nasal cavity with NQO1 (red) and OCAM (green) with DRAQ5 nuclear staining (blue). Scale bar in (**B**) indicates 500 µm.

The ciliated columnar cells that are found in the respiratory epithelium have many cilia (~300) to remove sticky mucus from respiratory surfaces, whereas the number of cilia that emerge from the dendrite of an olfactory sensory neuron is relatively small, 5 to 30, and the olfactory cilia are almost immotile. The membrane of olfactory cilia houses olfactory receptor proteins. Odorant molecules that are inhaled when we breathe, bind to these olfactory receptor proteins, thereby transducing odorant molecules into intracellular signals which activate olfactory sensory neurons. Olfactory receptor proteins form a large gene family of G-protein coupled receptors that are expressed in the olfactory epithelium [102,103,104,105]. There are more than 1000 genes in the mammalian genome that encode the many different olfactory receptor proteins. However, not all of them are expressed and functional. In mice, 1400 genes are found in this olfactory receptor multigene family, whereas the gene family consists of around 400 functional and 600 pseudogenes in humans [106,107,108,109]. Despite the large number of olfactory receptor genes in the genome, a given olfactory sensory neuron expresses only one of them (one olfactory sensory neuron—one olfactory receptor rule) [102,110] (Figure 3A). The olfactory epithelium houses several million olfactory sensory neurons. The ones that express the same olfactory receptor project their axon to the same one or two glomeruli in the olfactory bulb, where the axon terminals form synaptic contacts onto central neurons. Moreover, the expression pattern of olfactory receptor genes presents itself as four different zones of the olfactory epithelium [111,112,113] such that olfactory sensory neurons that express the same olfactory receptor are found in only one of the four zones. Furthermore, the dorsal zone (Zone 1) and the three other zones (Zone 2 to 4) were found to have differences in the expression of the neural cell adhesion molecule known as olfactory cell adhesion molecule (OCAM) [114]. It was not expressed in the dorsal zone and only expressed in the rest of the zones, Zone 2 to 4 (Figure 3B).

In the olfactory bulb, sensory information coming from the nose is initially processed in olfactory glomeruli. In the mouse, about 2000 glomeruli are present in each of the two olfactory bulbs. Though a single glomerulus receives massive axonal projections from olfactory sensory neurons, those neurons express a given odorant receptor. Thus, a single glomerulus represents odor information derived from only a given olfactory receptor (one glomerular-one olfactory receptor rule [115,116]). The glomeruli in the olfactory bulbs are organized chemotopically [117,118], such that a glomerulus is a discrete functional unit and serves as an anatomical address to collect and process specific molecular features about the olfactory environment, conveyed to it by olfactory sensory neuron axons expressing specific olfactory receptor proteins [119,120,121]. Each glomerulus has a shell of interneurons and glial cells [122], inside of which the dendrites of interneurons and output neurons receive olfactory sensory neuron input [123,124,125,126]. The glomerular interneurons are collectively termed juxtaglomerular cells and include periglomerular cells, short-axon cells, and external tufted cells [123,124,127,128]. Olfactory sensory neuron axons also synapse on output neurons, the mitral/tufted cells. Twenty to fifty mitral/tufted cells innervate each glomerulus and project their axons out of the olfactory bulb. Because one mitral/tufted cell has only one primary (apical) dendrite which projects to a glomerulus, one mitral/tufted cell receives excitatory synaptic input derived from one glomerulus, thus from one olfactory sensory neuron. A mitral/tufted cell has several secondary dendrites which extend horizontally in the external plexiform layer of the olfactory bulb. The secondary dendrites make dendro-dendritic synaptic connections with granule cells, the major inhibitory interneurons in the olfactory bulb. Thus, the response of a mitral/tufted cell basically reflects the sensory input from a given olfactory sensory neuron, but the response is shaped by inhibitory input from granule cells. Just as a glomerulus is a functional address for specific odorant features, mitral cells that innervate a specific glomerulus typically respond to a specific set of odorants. A given odorant can activate mitral cells in several or many glomeruli. Odorant identity is determined by the olfactory sensory neurons that are activated in the olfactory epithelium in response to odor stimulation. An odor is encoded through the combination of activated olfactory sensory neurons, where each olfactory receptor detects a molecular feature of the odorant [129].

Mitral/tufted cells connect the olfactory bulb with higher order brain centers for processing of olfactory signals [130]. The axons of mitral/tufted cells run in the lateral olfactory tract and terminate in olfactory centers on the ipsilateral brain side. The projection targets include the anterior olfactory nucleus, tenia tecta, olfactory tubercle, nucleus of lateral olfactory tract, piriform cortex, lateral amygdaloid complex, and entorhinal cortex. The olfactory pathway sends sensory information directly from the olfactory bulb to cortical centers [127,131,132]. A large number of centrifugal axons originate, in higher olfactory centers, and provide modulatory feedback to inhibitory interneurons [132,133,134]. In addition to the feedback input from olfactory cortices, centrifugal fibers originating in the basal forebrain (horizontal limb of the diagonal band of Broca, cholinergic fibers) and midbrain (locus coeruleus, noradrenergic fibers, and raphe nucleus, serotonergic fibers) could mediate olfactory processing during different behavioral states [135,136,137,138]. The centrifugal fibers arrive in the olfactory bulb by way of the anterior olfactory nucleus and the anterior commissure, rather than the lateral olfactory tract [132,139,140,141].

**Figure 4:**
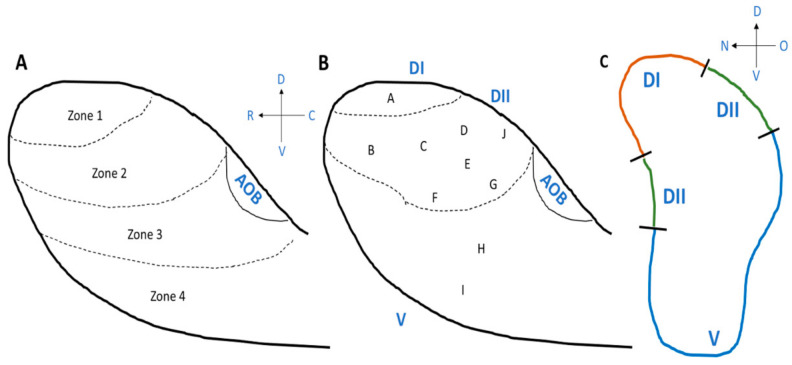
(**A**) Zone distribution in the olfactory bulb based on the regions where axons from the olfactory sensory neurons reach. (**B**) Odor Cluster distribution in the olfactory bulb based on Mori et al. (2006) [143] and Mori and Sakano (2011) [144]. (**C**) Domains of Odor Clusters shown in coronal section shape of olfactory bulb. AOB: accessory olfactory bulb. DI: Domain 1, DII: Domain 2, V: Ventral region, D, V, R, C around the arrows indicates dorsal, ventral, rostral, caudal, respectively, and D, V, N, O around the arrows indicates dorsal, ventral, nasal septum side, and outer side, respectively.

Starting from the discovery of the olfactory receptor genes [102], we learned that we detect and distinguish odors (odorous chemical compounds) in the environment (over 10^12^ odorant chemical compounds) using a large number of olfactory receptors. Studies using mice as animal models have shown that, in the olfactory bulb, there are four different zones, Zone 1 to Zone 4 from the dorsal region to the ventral region of the olfactory bulb (Figure 4A) and the locations of the olfactory sensory neurons, which project their axons, are also distributed in zone-specific ways in the main olfactory epithelium, from the dorsal area to lateral/ventral areas, as described above [114,142]. Importantly, there are domain-dependent differences in the odorants that activate the glomeruli [143,144] (Figure 4B,C). The odors are classified into Clusters A to I in the olfactory bulb [143] (Figure 4B). The most dorsal domain (DI) of the olfactory bulb is where odor Cluster A is located, and glomeruli are activated by amine and fatty acid chemical compounds. Beneath the most dorsal area is an area called DII, which is located between DI and the ventral domain. Odor Clusters B (aliphatic alcohols), C (phenol family odorants), D (variety of ketones), and J activate the glomeruli in DII. Odors included in Cluster J are trimethyl-thiazoline (TMT) and various pheromones (for example, 2-sec-tutyl-dihydrothiazole (SBT) and dehydro-exo-brevicomine (DHB) and other male urine odorants) [144]. The odors detected in the ventral domain, which includes odor Clusters E, F, G, H, and I, are methoxypyrazines, green odorants, C6 and C9 compounds, isothiocyanates, terpene hydrocarbons, esters, terpene alcohols, and sulfides (foods, fruits, and vegetables) (Figure 4B). It is still not clear whether humans have the same zone structure in the olfactory epithelium and olfactory bulb, such as the one found in mice. If so, these studies suggest that the area where, for example, terpenes in essential oils are sensed in the olfactory epithelium could be the lateral/ventral areas and that the lateral to ventral domain in the olfactory bulb could be the area where the glomeruli become activated by terpenes.

### 3.2. What Determines How the Odors Smell?

Two passages exist for odor stimulation. In one passage, odorant molecules find their way to the olfactory sensory neurons through the nose (orthonasal stimulation). In the second passage, odor molecules that enter the mouth during eating or drinking, travel from the mouth to the nose via the back of the throat and stimulate olfactory sensory neurons upon exhalation (retronasal stimulation) [131]. Retronasal olfactory stimulation can be confused with taste, which takes place in taste buds in the tongue and soft palate of the oral cavity. Food odors and the consistency of the food (“crunchiness”) together with tastants contribute to the flavor or aroma of food.

The roughly 400 different olfactory receptors in the case of humans contribute to the detection of volatile chemical compounds, which become perceived as odors. Odors of, for example flowers, extracts of herbal plants, and food can be constructed by a large number of different chemical compounds and perceived as “the odor of X”, i.e., odor of a thing X is in most cases not generated by a single chemical compound but rather by a group of many different chemical compounds. The concept of how odors are perceived was explained as being a result of certain combinations of these chemical compounds [144]. However, there have been studies from even before these findings that there are some individual differences in the way odors are detected, suggesting that some factors, such as genetic differences or environmental differences, may affect the way odors are perceived [145].

#### 3.2.1. Environment, Experience and Epigenetic Influences on Olfactory Receptor Gene Expression

Scientific studies using animal models have found various factors that affect the olfactory system, for example, olfactory fear conditioning, learning, epigenetic changes, the stage in the estrous cycle, and social environment. Depending on the type of odorants, exposure/lack of exposure to odorants in the environment has opposite influences. In the case of pheromones, the lack of odor enhances the sensitivity to them [146]. Responses to pheromones are affected by estrous cycle status in female mice in a way that, during the diestrus stage, the vomeronasal sensory neurons are silenced, and start responding to male pheromones while the females are in estrous stage, and these silencing effects were found to be mediated by progesterone [147,148].

When the odorants are non-pheromonal, the influence of exposure or lack of exposure becomes different. Increased exposure to odors is found to stimulate the birth of the olfactory sensory neurons [149]. When mice were exposed to a specific odor when they experienced fear, the olfactory receptors for specific odorants increased and they became more sensitive to the odor, showing avoidance at a lower concentration of the odor [150,151]. In addition, when male mice were used in this fear conditioning, and mated with naïve females, the offspring showed higher sensitivity to the odor without any fear conditioning to the odor and without spending time with the sire [151]. These trans-generational influences of fear-conditioned olfactory sense were mediated by epigenetics through the sperm of the sire [151]. These changes in olfactory sensitivity were generated by fearful experiences accompanied by an odor but this can happen by rewarding appetitive conditioning as well, producing a larger number of olfactory sensory neurons and larger glomeruli [150] and also by repeated exposure [152].

These studies using animal models indicate that exposure to odorants can stimulate an increase in the sensitivity to odors and an increase in the number of new olfactory sensory neurons for non-pheromone odorants, supporting the effects of smell training, and that sensory neurons for pheromones are regulated by different mechanisms from those for non-pheromones.

#### 3.2.2. Modulation at the Olfactory Epithelium and at the Olfactory Bulb

Most odors, such as the smell of rose, lavender, and foods, are not a single chemical compound. They are mostly composed of a large number of chemical compounds. In earlier years, when odors were found to be detected by hundreds of different types of olfactory receptors for different types of chemical compounds, it was considered that a smell that we perceive is determined by the combination of different, activated types of olfactory receptors, which transfers the signaling to the olfactory bulb and then to the brain. Recently, however, it was found not to be that simple. When olfactory sensory neurons are exposed to a mixture of multiple types of odorants, for example type a, b, and c, the responses did not become “a + b + c”. The odor type “a” rather became enhanced to “A” or suppressed to “_a_” [153]. This reminds us of the fact that often sensory neurons do not detect everything in the environment, as we often experience with our vision. The mechanisms of these modulations of enhancement or suppression are yet to be determined. Whether these sophisticated system modulations in the responses of olfactory sensory neurons are reestablished in regenerated olfactory epithelium could be one of the reasons for the occurrence of distorted smell, parosmia, which often happens after regeneration of olfactory sensory neurons following damage.

Another aspect in relation to non-equivalent roles of the chemical constituents of the odors is the order that glomeruli in the olfactory bulb become activated. Using an optogenetic approach with an animal model to activate the glomeruli of a specific region in the olfactory bulb in a specific order, it was found that the glomeruli activated earlier had larger effects on the behavioral responses. This suggested that, other than the enhancement/suppression at the peripheral region (olfactory sensory neurons), how the smell is perceived is affected by the way glomeruli are sequentially activated in the olfactory bulb. The reason for these sequential differences in the activation of glomeruli has not been determined yet, but studies using natural olfactory stimuli have also observed the sequential differences in the activation of glomeruli following exposure to various natural odors [154].

#### 3.2.3. Genetic Variation and Smell

As Wysocki and Beauchamp (1984) [145] proposed in earlier years, there are genetic variations that affect the way odors are perceived. A variant of olfactory receptor OR7D4 (WM/WM), which has just two changes in the amino acids, R8W and T133M (OR7D4, RT/RT), had less sensitivity to the ligand odorants androstenone and androstadienone [155]. In addition, the sensed smell was perceived as less pleasant by the genotype RT/RT of OR7D4 compared to the genotype RT/WM and WM/WM. There are several other olfactory receptor genes known to have variants, for example, OR11H7P [156] (isovaleric acid), OR2J3 [157] (*cis*-3-hexen-1-ol), OR5A1 [158] (ß-ionone), OR10G4 [108] (guaiacol). Polymorphism in olfactory receptors was found in about 63% of the olfactory receptors [108]. More recently, thorough investigation of gene expression and its influences on sensitivity to odors and to the perceived pleasantness was conducted [159]. These studies have found that the genetic variation reduces the function of the olfactory receptor, which enhances or reduces the pleasantness depending on the olfactory receptor type. These reduced functions in the olfactory receptor from genetic variation were associated with reduced perception of intensity of the odor, which was separate from the threshold concentration, that is, the “genetic variation in a single receptor had a greater effect on intensity and pleasantness than on detection threshold” [159]. There were also sex differences in the olfactory acuteness [152]. As written above, frequent exposures to an odorant increase the sensitivity to the odor, but these effects of enhanced sensitivity by frequent exposure to odors were found to be stronger in females than in males [152].

## 4. Smell Training to Enhance the Recovery of Olfactory Sense

### 4.1. The History of Smell Training

Olfactory dysfunction can be divided into two major categories: one is a conductive olfactory loss, which is caused by disturbances of the airflow to the olfactory mucosa, and the other is a sensorineural olfactory loss, which is caused by damage to the olfactory neuroepithelium and central olfactory pathway [160]. In the former case, treatment of the mucosal edema caused by rhinosinusitis improves olfactory dysfunction. In contrast, no evidence-based medical treatment for sensorineural olfactory loss has been developed. Many types of drugs, such as zinc preparations, Chinese medicine, topical and systemic steroids, vitamins, and metabolic agents have been tested, but none of them have been shown to be effective in placebo-controlled randomized controlled trials [161].

In 2009, Hummel et al. reported that olfactory training using odorants was effective in improving sensorineural olfactory loss. In their study, 56 patients with sensorineural olfactory loss (PVOD, traumatic, and idiopathic) were divided into two groups: one group did olfactory training with four odorants (phenylethyl alcohol (rose), eucalyptol (eucalyptus), citronellal (lemon), and eugenol (clove) twice a day for 12 weeks. The four training odorants were selected based on the classical classification of primary odors (odor prisms) proposed by Henning in 1916. The other group of patients did not do such olfactory training. Sniffin’ Sticks tests before and after the intervention period revealed that the training group showed better improvement of olfactory function [13].

Since then, various protocols have been used to study olfactory training. For example, a comparison of 16 weeks and 56 weeks of training showed a greater improvement in the latter, suggesting that long-term stimulation is recommended [162]. As for the training method, a multicenter randomized crossover study in Germany reported that stimulation with high concentrations of olfactory elements was more effective than training with low concentrations of olfactory elements [163]. It has also been reported that changing the types of odors periodically during olfactory training can enhance the success rate [164]. A recently published meta-analysis showed that patients with PVOD who received olfactory training had a 2.77 higher odds of achieving a clinically important difference in Sniffin’ Sticks Score compared to the control [165]. Another meta-analysis [166] showed that olfactory training had a small effect on olfactory threshold, but a significant effect on olfactory discrimination and olfactory identification.

### 4.2. Using Odorants for the Stimulation of Olfactory Neurogenesis

Neurogenesis continues throughout life. There are two major locations in the brain involved in adult neurogenesis, one is at the subventricular zone (SVZ) and the other is at the subgranular zone (SGZ) of the dentate gyrus (DG). The neuronal precursor cells born at the SVZ migrate a long distance, through the rostral migratory stream (RMS) to the olfactory bulb and differentiate into interneurons [167,168]. The RMS itself, as an extension of the SVZ, is also a niche for neurogenesis [169,170], and some of the neural stem cells born at RMS migrate to the olfactory bulb and become integrated as interneurons there, whereas some remain in the RMS and become glial cells [170]. The cells born at the SGZ migrate a short distance and become interneurons at the hippocampus. Neurogenesis continues at peripheral locations as well, such as at the olfactory epithelium where the olfactory sensory neurons are replaced periodically. The olfactory system is thus maintained by continuous turnovers of the olfactory sensory neurons and the interneurons in the olfactory bulb.

Age affects the rate of neurogenesis. It is high at early developmental stages, and, in the case of mice, the rate drops to the adult level of olfactory neurogenesis at one month old, which is pre puberty in male mice and post-puberty in female mice, and is maintained at this rate throughout adulthood [100,171]. The rate decreases at the senescence stage, although it does not totally stop [100,171]. When the olfactory bulb is deprived of sensory input by removal of olfactory sensory neurons or naris closure, apoptosis takes place at the SVZ as well as at the RMS first, which is followed by an increase of cell proliferation at the SVZ and RMS (for SVZ, [172,173]; for RMS, [170]). When the olfactory epithelium is damaged, age-dependent differences in the recovery of olfactory sensory neurons are observed [174]. When damage was experimentally generated in mice in the olfactory epithelium at an early developmental stage, the stem cells started to appear from as early as post-injury day 4, and mature olfactory sensory neurons (measured by olfactory marker protein; OMP+) started to appear as early as post-injury day 7 [174]. In the case of adult mice and senescent mice, the time process was similar but the number of new cells following the injury was much less and histological recovery was especially reduced in the senescent mice [174].

Recent studies using mice as an animal model have shown that there are specific subtypes of olfactory sensory neurons (or receptor genes) that are sensory input dependent/independent [149]. Nasal closure reduced the number of olfactory sensory neurons with specific receptor genes (input dependent) but there were olfactory sensory neurons with other specific receptor genes that did not change in number (input independent). Lack of olfactory stimulus due to nasal closure affected the production of *new* olfactory sensory neurons with the sensory input dependent type of receptor genes negatively but did not change the production of *new* olfactory sensory neurons with the sensory input independent type [149].

Various factors have been reported to affect the rate of neurogenesis [167,168]. Exposure to odors affects neurogenesis both at the olfactory epithelium [171] and at the SVZ [170,175] and RMS [170,176]. The effect of exposure to odors on neurogenesis at the SVZ is significantly stronger when different odors were used at each time of exposure than when the odors used were the same [177]. This was not due to the number of odors the subjects were exposed to. In the experimental setting where animals were exposed to the same odor daily, the number of the odor types was the same [177]. When neurogenesis in the SVZ was enhanced by enriched odor exposure, memorization of the odors was enhanced [175,177,178]. Dopaminergic interneurons were specifically enhanced in the olfactory bulb by enhanced neurogenesis caused by exposure to odors, suggesting their critical role in the neural circuit for olfactory information [179].

The types of odors used in the studies of neurogenesis in the SVZ are from foods and herbs [170,175,177] and pheromones of the opposite sex [180,181,182,183]. Table 1 shows examples of the studies using non-pheromone odorants/aromas for odor enrichment studies in animal models. The list of these odorants shows that the odors that stimulate neurogenesis do not need to have social meaning, and do not necessarily have positive or negative behavioral meaning for mice. However, neurogenesis in the peripheral system, i.e., the olfactory epithelium, involves various factors that cause differences in the impact of exposure to odors.

Utilizing genetic markers to specific olfactory receptor genes in mice, details of the effects of exposure to odorants on olfactory sensory neurons have been determined. IRES-tauLacZ is a transgene that will express the LacZ gene, which encodes ß-galactosidase, along the axons. When IRES-tauLacZ was tagged to the M71 murine olfactory receptor gene and mice were trained to discriminate acetophenone (ligand for M71), the axon density was higher and glomeruli size was larger in the mice trained with negative reinforcement using electric shock as well as in the mice trained with positive reinforcement using cocaine [150]. The number of olfactory sensory neurons with the M71 olfactory receptor were also significantly increased [150]. Interestingly, when mice were exposed without reinforcement, the glomeruli sizes were not different [150]. Studies have shown that odorant stimulation enhances the survival of the olfactory sensory neurons [184], and that the olfactory specific protein H2BE [185] and endothelin [186] are involved in activity dependent changes in the survival rate. This suggests that the increased number of olfactory sensory neurons following exposure to odors shown by Jones et al. [150] could be due to either or both the prolonged survival of the olfactory sensory neurons and/or enhanced peripheral olfactory neurogenesis. Jones et al. [150] also showed that, although there are many studies showing that odor enrichment enhances neurogenesis in the SVZ, for peripheral olfactory neurogenesis, it is important that odors have a meaning, whether it is negative or positive, in order to impact the number of olfactory sensory neurons. Importantly, such influences on the number of axons and glomeruli size were found to have trans-generational influences as well when using a negative reinforcement paradigm [151]. In addition, to complicate things further, recent studies have shown that there are separate subtypes of olfactory receptors, to which exposure to odors has different/opposite influences: for example, when olfactory sensory neurons with murine receptor MOR23 and M71 were exposed to lyral, the ligand of MOR23, and acetophenone, the ligand of M71, the olfactory sensory neurons with MOR23 decreased their density when they were exposed to lyral, whereas the ones with M71 receptors did not decrease their density when they were exposed to acetophenone [187]. This decrease in density, however, did not negatively affect sensitivity to lyral, but, on the contrary, they became more sensitive to the odor, and exposure to acetophenone did not change the sensitivity of olfactory sensory neurons with M71 receptors [187]. Exposure to odors thus has differential influences on the expression of the olfactory sensory neurons with receptors for these odors but these influences depend on the type of receptor [187,188].

The positive impact of exposure to odors was observed under the condition of recovery from olfactory dysfunction as well. When rats went through olfactory training for one week after dysfunction of olfaction due to infusion of *N*-methyl-D-aspartate (NMDA), they were able to distinguish the odors of cinnamon and vanilla [189]. When one nostril was occluded to block sensory input on one side after ablation of olfactory sensory neurons of both sides, regeneration of the sensory neurons on the occluded side and the open side were not different during the first weeks, but then the occluded side showed higher apoptosis, resulting in fewer mature olfactory sensory neurons during regeneration on the occluded side [190]. However, as written above, there are differences in the regeneration of olfactory sensory neurons depending on the types of receptors [149].

In summary, these studies on animal models suggest that (1) exposure to odors affects neurogenesis at the SVZ/RMS, which become interneurons in the olfactory bulb, and in the olfactory epithelium, (2) the influence of exposure to odors is not the same at the SVZ/RMS and at the olfactory epithelium, (3) these results suggest that smell training enhances regeneration and recovery of the olfactory sense in humans, (4) various types of odorants can be used in smell training but some may not have a positive influence in the case of peripheral olfactory epithelium neurogenesis, and (5) at a younger age, regeneration and recovery of the olfactory sense can take place faster.

**Table 1:** Examples of odors and procedures used for odor exposure studies using mice.

Olfactory Exposure Sources	Method	References
Lavender, garlic, paprika, marjoram, curry, rosemary, nutmeg, thyme, basil leaves, cumin, cardamom, tarragon, whole cloves, chocolate, celery, anise, ginger, lemon, orange, banana.	Exposed daily for 24 h to different odors placed in a tea ball hanging from the filter cover of the cages. Exposure days: 20 days or 40 days [175], 42 days [191], 31 days or 63 days or 42 days of enrichment + 21 days of standard condition [179]. Using mice.	[175,179,191]
Twenty different odors: pepper, star anise, fennel, cinnamon, garlic, onion, ginger, juniper berries, clove, nutmeg, lemon, celery, cumin, chocolate, cardamom, thyme, tarragon, capsicum, lavender, orange.	Daily renewal condition: the same sequence of exposure continued 20 days. Odor source was placed in a tea ball hung from the cage lid for 24 h. Same odor condition: 20 odors were mixed and presented. Odor source was replaced every 2 days. Using mice.	[177]
Lemon oil, juniper oil, clove oil, mint oil, lavender, musk, rosemary oil, tangerine, orange, sandalwood oil, thyme oil, sage, eucalyptus oil, cinnamon, calamint.	Twice a day, odor containing swab in a tea strainer placed on the cover of the cage for 1 h. Odorants selected randomly daily. Using rats.	[176]
Acetophenone, amazonica, dill, balsamic vinegar, basil, cocoa, (+)-carvon, cedar, cheese, chives, cineol, cinnamon, cloves, coffee, cumin, curry, “deodorant granules envirofresh apple, floral, lemon, and peach”, garlic geraniol, geranium golden wattle, hexanol, “honey and lemon cream”, isoamylacetate, juniper berries, lavender oil, linalool, (−)-limonelle, (+)-limonelle, lyral, massale, menthe piperite, mix morocco tea, nutmeg, olive oil, onion, oregano, paprika, Provence herbs, rosa, shallots, soybean sauce, strawberry, tarragon, tandoori, tobacco, vanilla, yeast extract.	“Odor pot” placed on the cage floor for 3 weeks, daily for 24 h to 3 different aromatic fragrances. Using mice.	[170]

### 4.3. Smell Training for the Suppression of Inflammation and Enhancing Regeneration

#### 4.3.1. Inflammation in the Damaged Olfactory Epithelium

Since the nasal cavity is exposed to the external environment, the olfactory mucosa can be damaged by a variety of agents such as viruses, bacteria, toxic chemicals, and allergens. These agents could directly insult the mucosa by their toxicity. Alternatively, they could induce activation of a host immune reaction which can cause secondary damage to the mucosa.

Olfactory disturbance in chronic rhinosinusitis is primarily attributed to a diminished airflow to the olfactory cleft, but in some cases olfactory function does not recover even after the maximum medical and surgical treatment to restore olfactory airflow. The previous papers suggest that a sensorineural degeneration is also involved in the pathophysiology of olfactory dysfunction in chronic rhinosinusitis [192,193]. In fact, transgenic mouse models of chronic rhinosinusitis, where TNF-α can be expressed in the olfactory epithelium in a temporally controlled manner, show disruption of the neuroepithelium when TNF-α expression was experimentally induced [34].

Inflammation is also involved in the pathogenesis of PVOD [23,24]. Intranasal administration of poly (i:c), a synthetic analog of viral double-stranded RNA, induced infiltration of inflammatory cells (neutrophils, lymphocytes, macrophages) in a time specific manner, upregulation of an inflammatory cytokine MIP2, and caused neuroepithelial damage [35]. Poly(i:c)-induced neuroepithelial damage was significantly inhibited by a neutrophil elastase inhibitor and was suppressed in neutropenic model mice, suggesting that the neutrophil-mediated innate immune responses may play an important role in the pathogenesis of PVOD.

Bowman’s gland, a secretory gland of the olfactory mucosa, contains a large quantity of metabolic enzymes comparable to that of the liver and takes substances from the blood and metabolizes them. When toxic metabolites are produced as intermediate metabolites, olfactory neuropathy occurs. Olfactory toxicity by the systemic administration of an anti-thyroid drug methimazole and a herbicide dichrobenil, which are often used as animal models of olfactory mucosal injury [194,195,196], is mediated through this mechanism.

The above-mentioned biological processes are considered to be an innate protective system, especially to protect the central nervous system, from foreign agents. However, they can also cause permanent olfactory damage. Therefore, it is important to regulate them with appropriate interventions in a clinical setting.

#### 4.3.2. Smell Training for the Enhancement of Regeneration

The olfactory neuroepithelium has a regenerative capacity. The basal cells of the neuroepithelium undergo continuous cell division to give rise to new olfactory neurons. When the neuroepithelium is injured, its regenerative ability is up-regulated and the epithelium is rapidly regenerated. The olfactory bulb also receives a constant influx of migrating neural progenitor cells from the subventricular zone [197]. These progenitor cells mostly become granule cells and form neural circuits with mitral cells and tufted cells, modifying the transmission of olfactory information. Despite this regenerative capacity, olfactory dysfunction often occurs in clinical settings, especially in the elder population, suggesting that such regenerative capacity could be deteriorated due to a pathological condition, such as inflammation and aging [36,198].

Recent studies have suggested that smell (olfactory) training has effects on these cellular dynamics. One study demonstrated that when methimazole was administered to mice to induce olfactory mucosal injury, followed by unilateral naris occlusion to block olfactory input, cell death in the closed side occurred more frequently than in the open side and neuroepithelial regeneration was incomplete [190]. Thus, it may be important to provide olfactory input to regenerating olfactory neurons in order to maintain their integration into existing neuronal circuits. Furthermore, in the olfactory bulb, the survival of nascent granule cells that migrated from the subventricular zone was reduced when the olfactory input was deprived in mice [199]. Thus, olfactory input may also contribute to the maintenance of neural circuits in the olfactory bulb.

Generally, sensory neurons depend on stimulus input for their survival, especially in the embryonic periods when the sensory neurons are overproduced and then selected for survival. This mechanism appears to be necessary to establish functional neural circuits with the appropriate number of neurons. Because the olfactory neural system retains capacity for continuous neural cell generation after birth, the cell fate specification of olfactory neurons may also be regulated by this principle, similar to neural tissues in the embryonic period.

## 5. Taste

### 5.1. Morphology of Taste Cells

**Figure 5:**
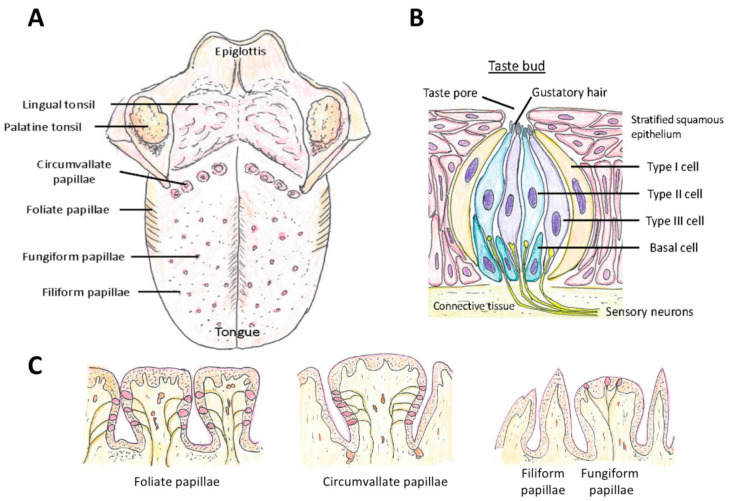
(**A**) Locations of circumvallate papillae, foliate papilae, fungiform papilae, and filiform papilae on the tongue. (**B**) Structure of the taste bud. The taste buds contain type I, type II, and type III cells as well as basal cells, which are the stem cells that become taste sensory cells or keratinocytes in the surrounding area. (**C**) Differences of the structure of the papillaes and the location of the taste buds in them (brown color circle).

Taste is sensed by the taste sensory cells (here we will call them taste sensory cells to compare them with olfactory sensory neurons. They are often called in different terms; for example, olfactory bud cells or taste cells), which are mainly located in the tongue (Figure 5A), but are found also in other locations in the oral cavity (palate, back of mouth, pharynx, epiglottis, and larynx) [200,201,202] (Figure 1). A very unique aspect of these taste sensory cells is that they form a bud-like structure, called a taste bud from their shape, which is comprised of 50 or 60 to 100 taste sensory cells [201] (Figure 5B). These taste buds are embedded in a specialized epithelium structure called a papilla. There are four types of papillae in the tongue classified by their shapes: the fungiform papillae, which are distributed broadly over the dorsal side of the tongue (Figure 5A,C) and usually contains one taste bud, the circumvallate papillae located on the posterior part of the dorsal surface of the tongue (Figure 5A,C), containing multiple taste buds, the foliate papillae which are located on the lateral parts of the tongue (Figure 5A,C), which appear as slits, containing several taste buds, and filiform papillae, which is not involved in sensing tastes (Figure 5A,C) [201,203,204]. Comparison of the tongues of various species suggests the evolutionary changes in the roles of tongue depending on the habitat of the species, from aquatic habitat to dry conditions, and the development of salivary glands [203]. Humans have more circumvallate papillae compared to rodents, which suggest a more developed taste sensing system, and rodents have harder keratinization of the epithelium over the dorsal tongue than humans, most likely because of the harder food they eat [203].

Another unique aspect of taste sensory cells is that they are specialized sensory cells that are not neurons. They arise from the stem cells at the base and outside of the papillae and not from neuronal progenitor cells (Basal cells in Figure 5B) [205,206,207]. Not all of these stem cells become taste sensory cells. Some become epithelial cells around the taste buds. They also do not extend axons, such as the olfactory and photoreceptor neurons [205], thus they are called short receptor cells. The tastants (taste provoking chemical compounds), other than sour tastants and salt, bind to the specific G protein-coupled receptors T1R, T2R, T3R (see below) expressed at the tip of the taste sensory cells (located at the Gustatory hair in Figure 5B). This activates the G-protein signaling cascade, which activates monovalent selective cation channel TRPM5 and causes depolarization in the taste sensory cell [201,208,209]. In short, in the case of taste sensing, the taste sensory cells generate action potentials, and not graded receptor potentials, in response to chemical stimuli, and release transmitters (ATP in the case of Type II cells and serotonin in the case of Type III cells) to activate gustatory afferent neurons, which are innervating the basolateral membranes of the taste sensory cells [202,208,209,210].

There are three morphologically classified types of taste sensory cells: Type I, Type II, and Type III (Figure 5B). The basic types of sensory perception by taste sensory cells are classified into sweet, salt, bitter, sour, and umami [211]. The roles of Type I cells are not fully known yet and considered to have a glia-like support function and they may be involved in salty taste sensing [201,202,209,210]. Type II cells are involved in sensing sweet, bitter, and umami taste, thus conducting the major roles in sensing the tastes [212]. They have the G protein-coupled taste receptors T1R and T2R, and T1R has three sub-members, T1R1, T1R2, and T1R3. These three sub-members form dimers in the plasma membrane with the combinations of T1R1 + T1R3 or T1R2 + T1R3.

#### 5.1.1. Sweetness

The T1R2 + T1R3 are involved in sensing sweet taste, which is generated by a broad range of chemical compounds: monosaccharides, disaccharides, some amino acids (for example glycine), and peptides as well as proteins (for example non-saccharide sweetener aspartame, i.e., the methyl ester of dipeptide L-aspartyl-L-phenylalanine), and some alcohols [213] (T1R3 homodimer can also sense sweet taste at high concentration [214]). The broad range of chemical compounds, with not only differences in chemical structure but also with large differences in molecular size, that can activate T1R2 + T1R3 type receptors bring questions on how they activate the same receptor [213,215,216]. Recent studies proposed that there are multiple binding pockets called Venus flytraps (VFT) that bind on the receptor with specificities to the different types and sizes of ligands [213,215,216].

#### 5.1.2. Umami

The dimers in the combination of T1R1 + T1R3 are involved in sensing umami. Compared to the broad range of chemical compounds involved in the sweet taste, the chemical compounds related to umami are more limited: glutamate, 5′-inosinate, and 5′-guanylate [217]. These chemical compounds generate the umami taste in a synergetic way rather than as a single chemical compound [217]. Metabotropic glutamate receptors (mGluRs), which are profoundly expressed in the central nervous system, are also expressed in the tongue, although their cDNA is shorter and thus are called taste-mGluR [218,219,220]. Specifically, taste-mGluR1 and taste-mGluR4 are expressed in the tongue tissue, taste-mGluR1 in the circumvallate papillae taste buds [221], and taste-mGluR4 in the foliate papillae taste buds [222] (Group II metabotropic glutamate receptors, mGluR2 and mGluR3 mRNAs are also found to be expressed in the circumvallate papillae taste buds [223]. Details on their roles have not been determined yet). Studies using T1R1 knockout mice and T1R3 knockout mice showed that these mice can still show responses to L-amino acids, the “umami” compounds (San Gabriel et al. 2009 [219] for T1R3; Choudhuri et al. 2016 [224] for both T1R1 and T1R3). Furthermore, studies using agonists/antagonists to mGluRs revealed that antagonists for mGluR1 and mGluR4 blocked the responses from inosine 5′ monophosphate and L-amino acids [224]. Overall, these studies have shown that T1R1 + T1R3 dimer receptors have significant roles in sensing umami taste, although mGluR receptors are also involved.

#### 5.1.3. Bitter

T2Rs (also known as TAS2R) are G protein-coupled receptors expressed on Type II cells and involved in sensing bitter taste. They are not co-expressed with T1Rs on the same Type II cells. Different from the small number of T1R genes found so far, there are 25 T2R genes found in humans and 36 of them in mice [213]. A broad range of chemical compounds are known as ligands of T2R [202,225,226]. Studies on T2Rs have found that, interestingly, they are expressed in various extraoral locations (Other than T2R, the T1Rs have also been found in extraoral locations: the gastrointestinal tract, brain, heart, liver and so on [202,227] and in skin (unpublished data, SK). The expression of sensory receptor genes expressed in cells located outside of the original tissues/organs is well known for the olfactory receptor genes, which are found in various tissue and organs as well as sperm cells, and thus not surprising. The roles of sensory cells are thus broader than they were first considered) [228,229]: airway epithelium, smooth muscle cells, human sinuses epithelium, and so on. Activation of T2Rs in, for example, the ciliated epithelial cells of airways and sinus epithelium make the ciliary beat frequency enhanced [229,230]. These studies indicate that the functions of T2Rs seem to be “protection” by sensing toxic substances by bitter taste in food and by enhancing ciliary movements [231]. There are studies suggesting utilization of T2R agonists in treatment of asthma and other diseases, including infectious diseases (for example, Nayak et al. 2019 [232]). The idea that, “good medicines are bitter” is now being supported by scientific data.

#### 5.1.4. Sour

The sour tastants directly activate acid-sensitive ion channels and initiate cation influx, which starts depolarization [208]. Depolarization activates voltage-gated sodium channels (SCN2A, SCN3A, and SCN9A) [208], which generates sodium influx, causing an action potential for the transmission of the signal.

Type III cells are involved in sensing sour tastes. There have been debates on the mechanisms used to perceive sour tastes for decades. Intracellular proton concentration (pH) is considered to contribute. Amiloride-sensitive epithelial sodium channels (ENaC) can serve as channels for entry, although they are found to be not solely responsible for the role [233]. Acetic acid (HAcetate; CH_3_COOH) and citric acid (H3Citrate; C_6_H_8_O_7_) permeate cells easily and release the protons [213]. Following other studies showing candidates of channels for entry (for example, PKD2L1 and PKD1L3 of TRP family channel), recently, studies have found that a proton-selective ion channel Otop1 is responsible for the taste of sour. Otop1 channels are expressed in Type III taste sensory cells, contribute as the entry path for protons, and are responsible for the action potentials generated to initiate the signaling to sense “sour” [234,235]. Transgenic mice without a functional Otop1 gene did not show responses to acids, indicating the role in perceiving sour taste [234,235].

#### 5.1.5. Saltiness

The taste sensory cells and receptors involved in sensing salty taste have been unclear [201,202]. There have been hypotheses on the involvement of Type I taste sensory cells through epithelial sodium channels (ENaC) [210], Type III taste sensory cells [236], and then in Type II taste sensory cells [237]. There is a possibility that all three types of taste sensory cells are involved in detecting salty taste. In a study using transgenic mice with Ca^2+^ indicator GCaMP3, exposure to NaCl generated responses in the Type II cells in fungiform taste buds. Many of the cells that responded to NaCl responded to saccharin as well (which reminds us of the well-known culinary tip that slightly adding salt enhances sweetness). In the same study, the Type III cells which responded to NaCl did not respond to citric acid and, ratio wise, over 80%, but not all, of the NaCl responsive cells were Type II cells [237]. In this study by Roebber et al. (2019) [237], Type I cells were not examined. Thorough studies are still necessary to determine the roles of each sensory cell type and the receptors involved.

### 5.2. Turnover of Taste Cells and Regeneration

Similar to the olfactory sensory neurons, taste sensory cells are renewed throughout one’s life and the longevity of the cells show individual and cell type (Type I, II, and III) differences. Using 5-ethynil-2′-deixyuridine (EdU) incorporation to label newly produced cells and to follow their fate, studies have found that the half-life of Type II cells was 8 days compared to the half-life of 22 days in the case of Type III cells [238]. Interestingly, Type I cells contained two groups with different half-lives, one (about 60 to 80% of Type I cells) with 8 days’ half-life and the other group (about 20% to 40% of Type I cells) with 24 days’ half-life [238]. A large number of new stem cells differentiate into Type I cells, whereas an intermediate number of cells differentiate into Type II cells, and the lowest number of progenitor cells differentiate into Type III cells. These stem cells are located at the bottom and outside of the taste buds. The location in the tongue also affects the expression of the type of taste sensor: the Type III cells, which are involved in sensing sour taste, are expressed more in the posterior location where circumvallate papillae are, compared to the receptors for sweet taste expressed in Type II cells, which are expressed more in the anterior location where fungiform papillae are [239].

The causations of ageusia/hypogeusia due to COVID-19 are not determined yet and it is possible that there are multiple causations involved (weakened signaling from inflammation and morphological damage caused by SARS-CoV-2 virus). If there is morphological damage in the epithelium of the tongue, it will go through a process of regeneration. In regular (non-regeneration) taste sensory cell turnovers, genes of Wnt/ß-catenin, bone morphogenetic proteins (*bmp*), sonic hedgehog (*shh*), fibroblast growth factors (*fgf*), and epidermal growth factor (*egf*) are involved in embryonic taste bud development [201,239]. Keratin 5 as well as keratin 14 (K5 + K14) genes are expressed in the basal keratinocyte progenitor cells and some, but not all, of these progenitor cells migrate to the bottom of the taste buds, and turn *shh*+. These *shh+* basal cells are post-mitotic precursors and not stem cells, and differentiate into the final type of cell [240]. Other progenitor cells differentiate into epithelial cells. Interestingly, when the SHH gene is expressed using genetic engineering techniques, the cells formed the typical onion-shaped taste buds instead of differentiating into epithelium cells [241], showing the major role of SHH in taste bud formation. During this process of renewal, when the fate, i.e., the type of cells, of the progenitor cells is determined has not been learned in detail yet. Recent studies, however, have shown that the Wnt/ß-catenin pathway seems to have a significant role in the fate of the cell types [242]. When ß-catenin was activated in *K14 + K5* progenitor cells (not *shh+* cells), almost all the cells differentiated into Type I taste sensory cells, some into Type II, and none into Type III cells [242]. When ß-catenin was activated in *shh+* precursor cells, the cells differentiated into Type I, Type II, and Type III in the ratio that is observed in usual taste buds [242]. This suggests the significant role of the Wnt/ß-catenin pathway in regulating the cell fates.

Regeneration starts after damage to the tissue/organ. In a recent study on a stem cell population in the tongue, leucine-rich repeat-containing G-protein-coupled receptor 5 (Lgr5) was found expressed in the cells at the base of circumvallate papillae and foliate papillae and became all three types of taste sensory cells [243]. When glossopharyngeal nerves, which innervate circumvallate papillae taste sensory cells, were surgically cut, LGR5+ stem cells were found to differentiate into all three types of taste sensory cells (Molecular markers for the taste progenitor cells/stem cells for each type of taste sensory cells have been found. Type I: GLAST, NTPDase2, antigen H; Type II: PLC-ß2, TRPM5, IP3R3, gustducin, Ggumma13, T1Rs, T2R2; Type III: NCAM, SNAP25, PKD2L1, AADC, serotonin (5-HT) [243,244] and carbonic anhydrase IV [245]) four weeks after surgery [245].

The wound healing process is classified into four overlapping stages: hemostasis, inflammation, cell proliferation and migration, and maturation and scarring [246,247]. When the regeneration process is initiated, there is a surge of proliferating cells around the wound bed. The cell proliferation rate is high in the area surrounding the edge of the wound in the case of cutaneous wound [16,248]. Keratinocyte stem cells migrate from these surrounding areas toward the wound bed and then toward the center of the wound bed [16,248,249]. Notably, in the case of skin, hair follicle bulge stem cells, which usually differentiate into hair, around the wound migrate to the epidermis and convert to epidermal stem cells temporarily [250]. Similar to stem cells in the tongue, Lgr5 is expressed especially in the lower hair follicle bulge stem cells [251,252,253]. The epidermis of the skin and oral epithelia have much in common [254]. The layer structure and the keratins expressed in each layer are also similar. It is possible that the agents that stimulate wound healing of skin may stimulate it in the oral epithelia as well.

### 5.3. ACE2 Expression in the Oral Cavity and Ageusia

The loss of the sense of taste is now well known as one of the major symptoms of COVID-19. Taste dysfunction is observed in almost 50% of the COVID-19 patients [11]. ACE2, which is one of the receptors that the virus binds to enter the cells, is expressed in the oral cavity, although there are some differences in the results of the studies, which conducted RNA sequencing and immunohistochemistry analyses. In a study using single cell RNA sequencing, many ACE2 genes were found expressed in the basal area of the epithelium around filiform papillae and a small amount were expressed in the Type III taste sensory cells [255]. In immunohistochemistry analyses, ACE2 and transmembrane protease serine 2 (TMPRSS2) were found strongly expressed in the taste buds of fungiform papillae [53,256]. ACE2 and TMPRSS2 were found expressed in the gingival tissues [256], palate [53], surface epithelial cells of the tongue [257], salivary glands [50,258], and exfoliated epithelia in saliva [257]. These studies show that, not only taste sensory cells, but also various parts in the oral cavity can become the entry location of the virus and contract the virus. Once they invade and replicate, it can cause inflammation in the local area, and then further cause morphological damage. The inflammation and the morphological damage could be the cause of the loss of the sense of taste, which suggests that essential oils that have anti-inflammatory and antioxidant effects can be the first candidates to test their effects on enhancing recovery of the sense of taste.

## 6. The Chemesthesis

In addition to the olfactory and gustatory senses, there is a less well-known type of perception of chemical compounds called chemesthesis. Various sensory channels are involved in chemesthesis and involvement of transient receptor potentials (TRP) channels is especially well-known. Although the TRP channels are expressed in various types of cells in the oronasal cavity, those expressed on the trigeminal nerves are considered to have a major role in sensing chemesthesis. The trigeminal nerve (also called the fifth cranial nerve) expands its endings into the face, the eyes, and the oronasal cavity. There are three major branches, the ophthalmic branch, which innervates towards the eyes and the nasal cavity, the maxillary branch, which innervates towards the face and nasal cavity, and the mandibular branch, which innervates towards the oral cavity.

Chemesthesis is known as the sense of irritation, pungency, cooling, warmth, burning, and pain, thus, it is considered a mostly negative type of sensing with the function of protection. Irritants inhaled into the nostril often induce apnea, bradycardia, vasoconstriction, and avoidance behaviors, which also suggest the role of chemesthesis in protection. Recent studies, however, have suggested that chemical sensing by TRP channels is also involved in the sense of taste [259,260] and smell [261]. These multiple roles of chemesthesis are maybe due to the fact that TRP channels are expressed in various types of cells in the oronasal cavity. Other than in the trigeminal nerve where TRPV1, TRPA1, 8 are expressed [262,263], they are expressed in the olfactory sensory neurons (TRPV1–4, TRPA1, TRPM5, 8 [261,264]), the supporting cells and basal cells of the olfactory epithelium (weaker than in the olfactory sensory neurons [264]), the taste buds (TRPM5, TRPP2 [262,263]), and the epithelial keratinocytes throughout the oronasal cavity (TRPA1, TRPV1, 3, 4, TRPM9 [262]). Activation of the olfactory sensory neurons can occur from the TRP channel instead of olfactory receptors [261] and a classic study by Doty et al. (1978) reported that most odorants evoked some sensation in anosmic subjects [265]. Studies have also shown there are also interactions between the olfactory sense and trigeminal activation [266,267] and the sense of taste and trigeminal activation [260]. Many of the chemical compounds that enter the oronasal cavity activate the TRP channels in addition to activating the olfactory receptors and/or taste cells, affecting the quality of the sense or causing the sense by themselves. This all indicates that the olfactory, the gustatory, and the chemesthesis systems comprise a complex system as a whole.

Chemesthesis dysfunction was reported in COVID-19 patients although the prevalence was not as high as anosmia and ageusia [11,268]. This suggested that ACE2 could be expressed in the trigeminal nerve or other receptor channels involved in chemesthesis. There are studies suggesting both possibilities: negative (Cooper et al. 2020 [57] based on RNA sequencing data on the trigeminal nerve of mice in Nguyen et al. 2017, 2019 [269,270]) and positive (Shiers et al. 2020 in human dorsal root ganglion (DRG) neurons) [271]. The latter study used a limited number of samples and the samples were not from the trigeminal nerve, and the variance of the results was high, so that it is hard to compare the studies. It is possible that, similarly to olfactory sensory neurons, in which ACE2 is not expressed, some morphological damage or weakening by inflammation is involved in chemesthesis dysfunction due to COVID-19.

## 7. Phytochemicals for COVID-19-Induced Anosmia and Ageusia

### 7.1. Phytochemicals with Anti-Inflammatory Effects to Enhance the Recovery of Olfactory Sense and Taste

The odor types used in smell training have been often selected from flowery (for example, rose), foul, fruity (for example, lemon), aromatic (for example, cloves), burned, and resinous odors (for example, eucalyptus) [13]. Essential oils of rose, lemon, cloves, and eucalyptus have thus become the four types most often used in smell training and have shown positive effects, improving the olfactory sense of the patients with anosmia and/or hyposmia. For example, Gellrich et al. (2018) [14] have used these four types of odors for smell training and have shown an increased volume in the limbic system and the thalamus in the brain. Altundag et al. (2015) [164] used more odor types in addition to these four types of odors and showed that the larger number of odor types did not improve the results. Both patients with hyposmia and anosmia showed improvements after smell training using these four types of odors, indicating that differences in the severity of dysfunction of the olfactory sense do not make a difference in the positive effects of smell training [272]. Le Bon et al. (2020) have shown that smell training combined with oral corticosteroid treatment significantly improved the sense of smell after the loss of it due to COVID-19 [273].

These odor types used in smell training are mostly selected based on the classic study by Henning published in 1916 on the “odor prism” [274]. Although studies have shown the effects on improving the olfactory sense, we do not know if these four odor types are the most efficient choices to improve the olfactory sense. The purpose of smell training so far has been to expose the less/mal/non-functioning olfactory sensory neurons to odorants to stimulate them and improve their function, rather than utilizing the bioactive properties of the chemical constituents of these oils. The lack of acknowledgement of the bioactive properties is rather striking, causing even claims by some clinicians that the bioactivity of the chemical constituents is “completely unsupported by any science” (personal communication to SK). If we choose the essential oils based on the scientific evidence of the bioactive properties and the mechanisms of actions of the major chemical constituents, we can select the essential oils based on what we know about the effects of their chemical constituents, and we can use them in the most effective way.

#### 7.1.1. CB2 Receptor

There are various terpenes with bioactive properties [17,275,276,277,278]. For example, ß-caryophyllene is a sesquiterpene included in copaiba, lavender, and various other herbs, and it is a ligand of cannabinoid receptor 2 (CB2) [279]. ß-caryophyllene stimulates the release of β-endorphin [280] and suppresses inflammatory nociception [281,282,283]. Exposure of ß-caryophyllene to cutaneous wounds activated the pathways involved in cell proliferation and cell migration, suppressed the pathways related to inflammation, and improved re-epithelialization (see 6.d for the changes in the pathways and genes) [16]. ß-caryophyllene has a smell, indicating that it activates the olfactory system other than the CB2 receptors. Exposure to ß-caryophyllene through the air did not produce enhanced re-epithelialization of cutaneous wounds, indicating its influences were not mediated through the olfactory system and none of the olfactory receptors expressed in skin were involved [16]. In vivo experiments using a CB2 antagonist with ß-caryophyllene and a CB2 agonist without ß-caryophyllene, and in vitro experiments using primary cells from CB2 knockout mice showed that CB2 receptors are involved. However, the results on gene expression of the TRP channels and the results of the in vitro experiments suggested that the effects may not be solely mediated through the CB2 receptors and suggested the involvement of TRP channels [16]. In addition to ß-caryophyllene, recent studies have shown that citral, a monoterpene included in for example lemongrass, is an agonist of CB2 and CB2 antagonist AM630 blocked the anti-inflammatory effects by citral. CB1 antagonist AM281 did not block the effects by citral [284].

#### 7.1.2. GABA and Sodium Channels

Linalool is a monoterpene included in many herbs. Recent studies using mice as an animal model have found that linalool has an anxiolytic effect, which is mediated by the olfactory system through γ-aminobutyric acid (GABA) transmission. Benzodiazepine-responsive GABA_A_ receptors were involved [19]. Intranasal application of linalool using rats showed that it has anti-inflammatory effects [21]. In vitro studies showed that linalool activates TRPA1 and TRPM8 [20,21,22], and the anti-inflammatory effects could be mediated by the TRP channels as well, other than through the activation of GABA_A_ receptors. Activation of GABA_A_ receptors as a mechanism for the analgesic and anti-inflammatory effects by terpenes was suggested by many other chemical compounds and essential oils (for example, carvacrol, isopulegol, pinocarveol, verbenol, and myrtenol in *Sideritis* all had the potential to activate GABA_A_ [285]). There are also studies showing that some terpenes suppress Na channels and activate GABA_A_ receptors (for example, methyl eugenol had inhibitory effects to Na_v_1.7 channels and activated GABA_A_ receptors in in vitro studies [18]). As Na channels are the source of excitatory currents for the nervous system and the muscles, suppression/inhibition of the Na channels was suggested as one of the mechanisms that gives some terpenes analgesic influences [18]. Methyl eugenol is a direct derivative of eugenol [286], which also has analgesic effects and suppresses the excitability of the sciatic nerve and the superior cervical ganglion neurons [287].

The roles of the GABAergic system and sodium channels in the brain in relation to essential oils have been studied in various oils (for review, Wang and Heinbockel 2018) [288]. The delivery/administration methods in in vivo studies vary depending on the studies and the effects also vary, some showing increases in GABAergic responses (for example, *Acorus gramineus* (grassy-leaved sweet flag; delivery by inhalation and oral), *Camellia sinensis* (tea plants; delivery by inhalation), *Cymbopogon citratus* (lemongrass; delivery by oral), *Nigella sativa* (fennel; oral)) and some showed a decrease in the GABA-induced currents (for example, *Melissa officinalis* (lemon balm; delivery by oral)) [288]. In vitro studies using α-asarone, which is a major chemical constituent of *Acorus tatarinowii*, a plant used in Chinese herbal medicine, showed that exposure of cells to *Acorus tatarinowii* in the culture media inhibited the Na_v_ 1.2 channel and exposure of olfactory bulb tissue inhibited firing of mitral cell neurons [288]. In vivo studies have also shown that *Acorus tatarinowii* administered by gavage to male Sprague–Dawley rats two hours before treadmill running made the time until exhaustion on the treadmill longer by suppressing the exercise-induced increase of 5-hydroxytryptamine (5-HT; serotonin) [289]. It also suppressed the exercise-induced increase of tryptophan hydroxylase 2 (TPH2) and the exercise-induced decrease of the serotonergic type 1B (5-HT1B) in the dorsal raphe of the brain [289].

#### 7.1.3. Potassium Channels

Geraniol, nerol, ß-citronellol, citral, and linalool had inhibitory effects on the Kv1.3 channel (88.48 ± 2.83%, 79.43 ± 3.96%, 78.46 ± 1.05%, 50.71 ± 4.82%, and 49.53 ± 164%, respectively) and exposure of CD3+ T cells to geraniol showed that it suppressed T cell proliferation and reduced the production of IL-2, TNF-α, and IFN-γ [290]. Geraniol and citronellol are the major chemical constituents of the geranium essential oil [291]. Nerol and linalool are also included in geranium oil, although the percentage is lower than geraniol and citronellol [291]. Exposure of a human colorectal adenocarcinoma cell line (HT-29) to citronellol and geraniol (at a concentration that is not cytotoxic) significantly down-regulated the expression of ACE2 and TMPRSS2. This suggests that they may become one of the candidates for anti-COVID-19 treatments [291]. ACE2 is included in the renin-angiotensin system (RAS), which is involved not only in regulating the cardiovascular system but also in local functions independently. It degrades angiotensin II, which is involved in “pro-inflammatory, proliferative and pro-fibrotic activities” as well as “reactive oxygen species (ROS) production, cell growth, apoptosis, cell migration and differentiation, extracellular matrix remodeling” [292]. Degradation of angiotensin II, producing angiotensin 1–7, is important in adjusting the balance of the level of angiotensin II [292,293]. Although blocking the entry of SARS-CoV-2 virus to host cells through ACE2 using protease inhibitors has been receiving attention [59,294,295], excessively strong blocking of it may cause negative influences on the regular activities of ACE2 as well. The use of essential oils that have both the function of suppressing the secretion of cytokines as well as suppressing the expression of ACE2 and TMPRSS2 could be one of the promising methods to prevent and treat COVID-19.

#### 7.1.4. TRP Channels

Various phytochemicals, not only terpenes but also flavonoids, activate transient receptor potential (TRP) channels (for review, see Premkumar 2014 [296]). For example, TRPA1 (transient potential channel ankyrin 1) (curcumin, cinnamaldehyde, nicotine, linalool, eugenol, and others), TRPV1 (transient potential channel vanilloid 1) (capsaicin, eugenol, camphor, gingerol, vanillin and others), TRPM8 (transient receptor potential melastatin) (1,8-cineole (eucalyptol), menthol), and TRPC6 (transient receptor potential canonical) (hyperforin) are activated by the chemical compounds shown here in parentheses [296]. The essential oil of *Citrus aurantium* (bitter orange, or daidai) activates TRPA1 [297]. Of the ten major chemical constituents, (+)-Limonene was included the most (over 95%), and osthole had the strongest effect in activating TRPA1. Other major chemical constituents were linalyl acetate, linalool, (+)-carvone, (−)-carvone, geranyl acetate, osthole, geranyl propionate, neryl acetate, and citral, and they all activated TRPA1 although not as strongly as osthole [297]. TRPA1 (transient receptor potential Ankyrin 1) is the only member of the TRPA sub-family of TRPA subfamily, and it is expressed in both the central and peripheral nervous systems as well as in various extra-neuronal tissues [298]. Although it was first considered involved in detecting “pain, cold, and itch” sensations, those expressed in extra-neuronal tissues are now known to be involved in regulatory and pro-inflammatory pathways [298]. TRPA1 is expressed in abundance in macrophages and T cells, and studies using TRPA1 agonists and antagonists showed that activation of TRPA1 expressed in macrophages has crucial anti-inflammatory effects [299]. TRPA1 expressed in human airway cells and airway sensory nerves released pro-inflammatory neuropeptide SP when TRPA1 was activated together with TRPV1 [299], indicating the possibility of differential influences depending on the types of cells that they are expressed in and/or combinations of the TRP channels activated.

The TRPM8 channel is found activated by, for example, menthol, cannabigerol, and 1,8-cineole. 1,8-Cineole is a major chemical constituent of eucalyptus species [300]. In vitro and in vivo studies have shown that 1,8-cineole suppressed the production of interleukin (IL)-1ß, tumor necrosis factor α (TNFα), leukotriene 4 (LTB4) and thromboxane B2 [275,278,301,302]. Studies using 1,8-cineole on patients with bronchial asthma showed that the anti-inflammatory action enabled reducing the usage of steroids as well [303]. Intraperitoneal injection of 1,8-cineole (eucalyptol) clearly suppressed the levels of IL-1ß, IL-6, and TNFα to as low a level as control groups in mice injected with complete Freund’s adjuvant (CFS) in the hind paw [302]. Interestingly, transgenic mice without functional TRPM8 channels (TRPM8 knockout mice) showed as high an inflammation level as the control group mice that received CFS and were treated with a vehicle (corn oil), indicating that the impact of suppressing proinflammatory cytokines by 1,8-cineole was mediated by the TRPM8 channel [302].

#### 7.1.5. Multiple Routes by a Single Chemical Compound

What we can see from these studies are that (1) a single type of terpene can activate multiple channels/receptors and the influences of these separate pathways could be different. This may be the reason that a single type of terpene has multiple impacts, i.e., anti-analgesic, anti-inflammatory, anti-microbial, anti-viral, anti-fungal, enhancing cell proliferation, enhancing cell migration, and so on. (2) A single terpene can activate multiple channels/receptors and generate a single influence. Whether this activation of multiple channels/receptors strengthens the influences or whether it is a necessary aspect in inducing the influences needs to be determined.

In general, the process of regeneration of damaged tissues starts from the inflammation stage, then proceeds to the cell proliferation/migration stage, and finishes with the maturation/remodeling stage [304]. In COVID-19-induced anosmia and hyposmia, it is possible that the inflammation and morphological damage in the olfactory epithelium induced by the virus is causing the malfunctions. A difference from the inflammation and morphological damage due to injuries could be that the viruses may be still actively infecting there and replicating in the area, which may keep the inflammation continuing and the damage may further expand. It could be important to use terpenes with anti-viral effects together with the ones with anti-inflammatory effects at least until there is no further viral infection.

In summary, it is important to understand and consider the bioactive properties of the chemical constituents of the essential oils when they are used in smell training. Many terpenes activate multiple pathways. It is important to know the major chemical constituents in the essential oils and to understand their effects as well as the pathways that they activate in order to utilize the essential oils in the most effective way. For example, the influence of ß-caryophyllene on re-epithelialization was not mediated by the olfactory system. The damaged area needs to be exposed to ß-caryophyllene directly to cause the enhanced regeneration by ß-caryophyllene. The anxiolytic effect by linalool was mediated by the olfactory system, and it is necessary for linalool to access the olfactory system to induce the anxiolytic effect. It is thus important to know the routes to utilize the essential oils in ways that match the purposes.

**Figure 6:**
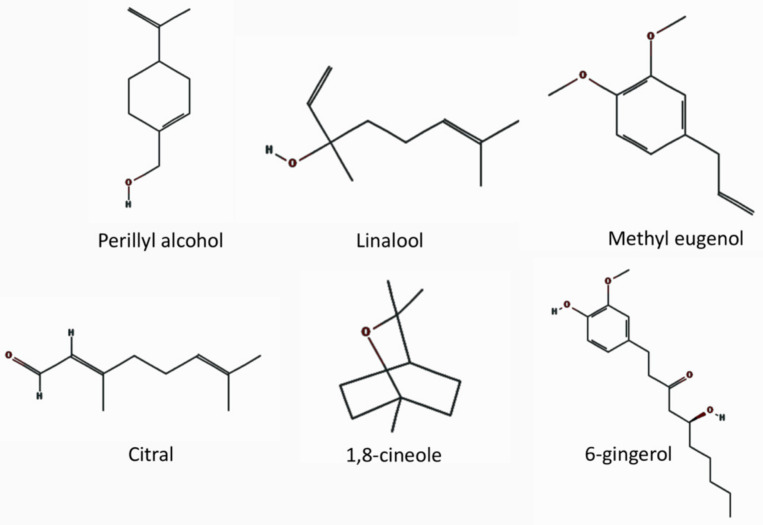
Chemical compounds with anti-inflammatory effects.

Although not covered here in detail, there are olfactory receptors expressed in skin as well. The influence of terpenes on these olfactory receptors outside the olfactory system are not well studied in detail yet, but there are studies suggesting possible involvement in wound healing and hair growth [305,306]. Table 2 shows essential oils with major chemical constituents that are known to have anti-inflammatory effects and Figure 6 shows examples of the chemical compounds with anti-inflammatory effects.

### 7.2. Phytochemicals to Enhance the Recovery of the Sense of Taste

Following the outbreak of COVID-19 caused by SARS-CoV-2, millions of people have lost their senses of taste and chemesthesis. Although it has already been decades since the smell training method using essential oils has been developed, there has been no “taste training” developed so far. Utilizing the scientific evidence on the bioactive properties of various chemical compounds, it could be possible to develop a “taste training”, i.e., a method and materials to facilitate the recovery of the sense of taste and chemesthesis. The development of “taste training” may significantly help the recovery of their senses. A difference from the olfactory system is that, in the case of the oral cavity, it is possible to include both volatile and non-volatile/low-volatile chemical compounds for the “taste training”.

#### 7.2.1. Terpenes

The essential oils with chemical constituents with anti-inflammatory effects that are shown in Table 2 can also be the candidates to test their effects on improving recovery from ageusia other than anosmia. It is specifically important to note the fact that the tongue is one of the specialized skins (others, for example, are nipples and lips, which have different morphological characteristics compared to regular skin and yet have common characteristics as well) with sensory cells to sense taste. In 2008, ß-caryophyllene, a sesquiterpene included in various herbs and spices such as lavender and black pepper, was found to be a cannabinoid receptor 2 (CB2) ligand [279]. Topical application of ß-caryophyllene on murine cutaneous wounds was found to improve re-epithelialization [16]. RNA sequencing of skin exposed to ß-caryophyllene has revealed that various epidermal stem cell marker genes were significantly up-regulated in skin applied with ß-caryophyllene (Gli1, Lgr5, Sox9, Lrig1) [16], and these genes are also involved in the tongue epidermal turnover. This suggests a possibility that utilization of ß-caryophyllene by mouth may stimulate the pathways related to epidermal stem cell proliferation in the tongue similar to what occurred in skin. In addition, pathways related to inflammation and the immune system (TREM1 signaling) were suppressed [16]. These studies suggest that ß-caryophyllene can be one of the strong candidates especially in facilitating regeneration and thus to facilitate the recovery of the sense of taste. We did not refer to this in the previous section on smell training because we see fewer similarities in the morphology of olfactory epithelium and skin.

#### 7.2.2. Flavonoids and Others

What is specific to taste is that it is possible to use non- and less-volatile phytochemicals for taste training. There are many chemical constituents with less volatility that could be included in diets as candidates to test their effects on improving recovery from ageusia. The candidate chemical compounds for anti-viral effects need to have high binding affinity to the virus as well as high selectivity to the virus (not cause damage to other cells/tissues). There are over 9000 chemical compounds in the group of flavonoids. Rather than attempting to cover broadly, we focus here on several of the edible plants and fruits, which are known with the phytochemicals to have anti-inflammatory effects.

### 7.3. Mechanisms of Anti-Inflammatory Effects

**Figure 7:**
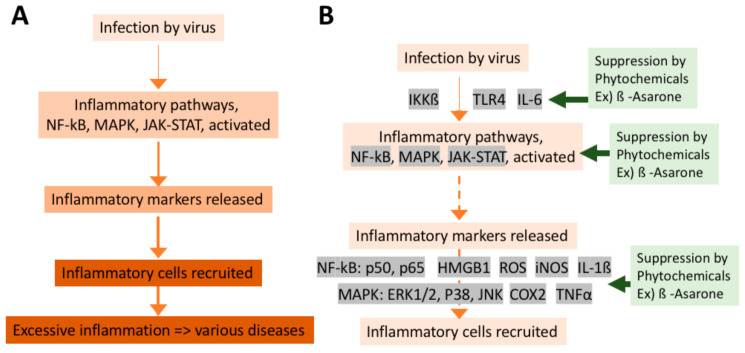
(**A**) Infection by virus stimulates the release of pro-inflammatory cytokines, activates inflammatory pathways, which stimulates the release of inflammatory cytokines/inflammatory markers. Intracellular signaling and nuclear translocation of molecules initiates transcriptional changes and changes in the expression of genes. Although studies have shown that inflammation is necessary for regeneration of olfactory epithelium, excessive inflammation and chronic inflammation can lead to autoimmune diseases, metabolic diseases, and cancer. (**B**) Studies have shown that the phytochemicals show effects to suppress the inflammation at each of the steps. This may have been caused either by their direct effects on each step or by the effects at the earlier steps that affects the later steps. Shaded molecules are the molecules in Table 2 that phytochemicals show suppression of secretion or expression.

**Figure 8:**
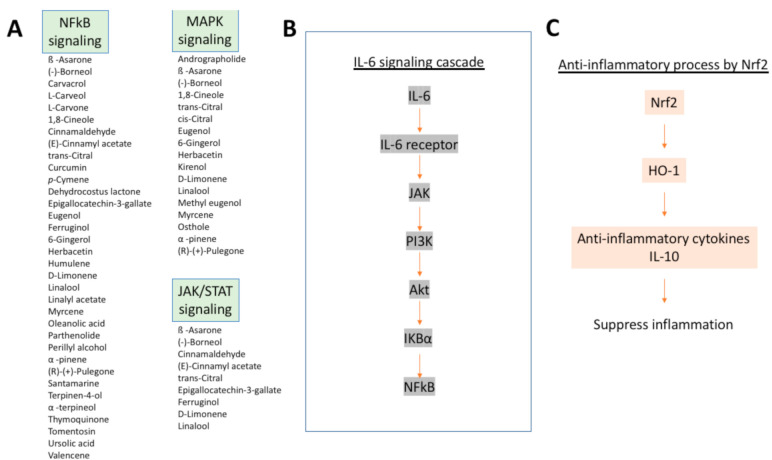
(**A**) Phytochemicals with suppressive influences on signaling pathways. (**B**) IL-6 signaling cascade through JAK/PI3K/Akt/IKBα/NFkB is suppressed at each step by phytochemicals, for example, Epigallocatechin-3-gallate, which is included in tea plant [345] (Table 2). (**C**) Cafestol enhances the expression of HO-1 and stimulates The Nrf2 pathway.1,8-Cineole, R-carvone, and luteolin enhance IL-10. Cannabigerol restores Nrf2 levels and linalool enhances nuclear Nrf-2 translocation (see Table 2 for details).

Many chemical compounds have been found to suppress pro-inflammatory cytokines and cell signaling pathways involved in inflammation (Table 2). Interleukins (IL) and tumor necrosis factors (TNF), for example, are secreted in response to inflammatory stimuli, such as infection by toxic substances/viruses/bacteria or injuries. Many chemical compounds in Table 2 show suppression of TNF-α, one of the major mediators of inflammation. TNF-α generates a positive feedback loop of TNF-α and other cytokines, and suppression of it can block the positive feedback. This will reduce the possibility of acute inflammation proceeding to chronic inflammation and/or auto-immunity and diseases caused by the progressed inflammation (Figure 7A). There are in vitro studies showing that S glycoprotein, but not membrane protein, envelope protein, and neucleocapsid protein, stimulated the secretion of inflammatory cytokines and chemokines, IL-6, IL-1ß, TNF-α, CXCL1, CLCL2, and CCL2 [307]. Inflammation was triggered by the S glycoprotein through activation of the NF-kB pathway [307]. Although inflammation is an important step in protection and important also in regulating the regeneration process in injuries [308,309], excessive inflammation and chronic inflammation can lead to severe conditions, fatal and/or life-long (Figure 7A) [308,310,311]. In the case of infectious diseases, virus replication causes strong inflammatory responses, which has been reported in many papers on COVID-19. Suppression of the excessive inflammation may facilitate recovery from the disease and the damages caused by the disease. Figure 7A summarizes some of the inflammatory signaling cascade, and Figure 7B shows the influences of some of the phytochemicals on the inflammation cascade (Figure 7B). Numerous phytochemicals suppressed the expression or secretion levels of pro-inflammatory markers (Table 2 and Figure 8B) and many of them specifically suppressed the NF-kB pathway (Figure 8A). IL-6 is a major pro-inflammatory cytokine that triggers multiple types of signaling cascades, and there are phytochemicals that suppress the molecules included in an IL-6 signaling cascade of IL-6/IL-6R/JAK/PI3K/AkT/IKBα/NFkB (Figure 7B). Importantly, some of the phytochemicals also enhance the secretion of anti-inflammatory cytokines, which are involved in suppressing excessive inflammation and stimulate the Nrf signaling pathway (Table 2 and Figure 8C). From the genes and proteins/peptides suppressed by the phytochemicals (Table 2), it is possible to say that there are many phytochemicals that can suppress the signaling pathways of NF-kB, and some of them also suppress the MAPK and JAK-STAT pathways. These results suggest that it will be highly beneficial to select the essential oils and diets by taking into consideration the chemical constituents and the targeted symptoms.

**Table 2:** Examples of phytochemicals known to have anti-inflammatory effects. Some papers cited did not have the information of the CID and CAS, and thus not all of the CID and CAS are based on the papers cited.

Chemical Compounds	Type	Effects (Suppress, Enhance); Receptors/Channels and Pathways Other than Olfactory Receptors, If Known	Examples of Source Essential Oils or Plants	References
Andrographolide CID: 5318517 CAS: 5508-58-7	Diterpene MW: 350.4	**Suppresses**: in vitro, Suppress NO, PLCγ2/PKC and PI3K/AKT-MAPK signaling pathways inhibiting platelet aggregation **Vapor pressure**: 1E-14 mmHg (25 °C)	Green chiretta (*Andrographis paniculata* (Burm. f))	[312]
Allicin CID: 65036 CAS: 539-86-6	MW: 162.3	**Suppresses**: in vivo, 50 mg/kgbw for 5 days in rabbits infected with Pasteurella multocida improved the inflammatory markers [313]	Garlic	[313,314]
Apigenin CID: 5280443 CAS: 520-36-5	Flavonone MW: 270.24	**Suppresses**: in vitro, NOD-like receptor family pyrin domain containing 3 (NLRP3) inflammasome action	parsley, celery, chamomile flowers	[315]
ß-Asarone CID: 5281758 CAS: 5273-86-9	Phen MW: 208.25	**Suppresses**: in vivo, suppress inflammatory cytokines, IL-6, IL-1 ß, iNOS, COX-2; suppress dizocilpine induced cognitive impairment; in vitro, suppressed LPS induced NO release, iNOS, COX-2, IL-1 ß, IL-6, TNFα, p65, lkBα, JNK, p38	*Acorus tatarinowii*, *Acorus gramineus*, *Asarum*, *Guatteria*	[316,317]
α-Bisabolol CID:1549992 CAS: 515-69-5	Sesq MW: 222.37	**Suppresses**: review, inhibition of leukotriene synthesis, leukotriene synthesis, 5-LOX; in vivo, in vitro	German chamomile (*Matricaria chamomilla*), Candeia (*Eremanthus erythropappus*), *Smyrniopsis aucheri*, Heartwood (*Vanillosmopsis pohlii*), sage (*Salvia runcinate*)	[276,318]
Bornyl acetate CID: 6448 CAS: 76-49-3	Mono MW: 196.29	**Suppresses**: IL-6, IL-8, MMP-1, MMP-13 **Vapor pressure**: 0.107 mmHg (25 °C)	*Amomum villosum*	[275,278]
(-)-Borneol CID: 64685 CAS: 10385-78-1	Mono MW: 154.25	**Suppresses**: IL-1ß, IL-6, TNF-α, CD16, CD206 expressions, IL-10, phosphorylation of NF-kB, IkBa, p38, JNK, TRPA1 mediated cationic currents **Enhances**: NO, iNOS enzymatic activity **Vapor pressure**: 5.02 × 10^−2^ mmHg (25 °C)	Sambong (*Blumea balsamifera*), Balkan heath, *Dryobalanops aromatica* (Borneol camphor), *Erica spiculifolia*; included in citrus peel oils, spices (nutmeg, ginger, thyme); skin irritation by contact; odor, such as camphor	[275,278,319]
Broussoflavonol B CID: 480828 CAS: 99217-70-6	Polyphenol MW:452.5	**Activates**: in vivo, in vitro, suppress pro-inflammatory responses by activating AMPK in 3T3-L1 adipocyte [320]	Paper mulberry (*Broussonetia papyrifera*)	[320]
Cafestol CID: 108052 CAS: 469-83-0	Diter MW: 316.40	**Suppresses**: review, PGE2, NO synthesis, COX2, iNOS, inhibit activation of inhibitor kB kinase **Enhances**: Nrf2/HO-1 pathway, increase the expression of HO-1	Coffee beans	[321]
Camphene CID: 6616 CAS: 79-92-5	Mono MW: 136.23	**Suppresses**: reduced nociceptive behavior **Vapor pressure**: 2.5 mmHg (25 °C)	*Cannabis sativa*	[322]
(+/−)-Camphor CID: 2537 CAS: 76-22-2	Mono MW: 152.23	**Suppresses**: **Receptors/Channels**: TRPV1 **Vapor pressure**: 0.65 mmHg (25 °C) Hazardous warning	Camphor tree (*Cinnamomum camphora*)	[278,296]
Cannabigerol CID: 5315659 CAS: 25654-31-3	Resorcinol MW:316.5	**Suppresses**: IL-1ß, TNF-α, IFN-γ, PPARγ, nitrotyrosine, SOD1, iNOS levels **Enhances**: restored Nrf-2 level **Receptors/Channels**: TRPM8 (some reports say TRPM8 antagonist), CB2, TRPA1, TRPV2, PPARγ, GPR55;	*Cannabis sativa*, non-psychotropic; partial agonist of CB2 but the affinity depends on the assays. CB1 unclear [323]; non-psychoactive	[323,324]
Capsaicin CID: 1548943 CAS: 404-86-4	MW: 305.4	**Suppresses**: in vivo, rat, significantly suppressed experimentally induced oedema due to egg albumin in the sub-plantar of the paw by IP injection of capsaicin (2 mL/kgbw)	Chili pepper	[325]
Carvacrol CID: 10364 CAS: 499-75-2	Mono MW: 150.22	**Suppresses**: reduced the activation of TLR4/NF-kB signaling pathway, suppressed IL-1ß, IL-6, IL-18, triglyceride, TNFα, suppressed levels of IKK, NALP3, NF-kB, TLR4, reduced p-IRS-1 and p-InsR levels (reduce insulin signaling molecules) in high glucose-induced HUVEC (human umbilical vein endothelial cells) **Receptor**: GABA_A_	*Sideritis*, oregano, thyme, pepperwort, wild bergamot	[275,285,326]
L-Carveol CID: 7438 CAS: 99-48-9	Mono MW: 152.23	**Suppresses**: in vivo, in vitro, in silico, suppressed blood glucose in alloxan-induced diabetic rats [327]; in vitro, NF-kB activity, TNFα, IL-1 ß, IL-10 [328]	Orange peel, dill, seeds of caraway (*Carum carvi*)	[275,327,328]
L-Carvone CID: 7439 CAS: 99-49-0	Mono MW: 150.22	**Suppresses**: NF-kB activity, TNFα, IL-1 ß, IL-10 (S-carvone; [329], IL-13 (S-carvone), IgE (S-carvone) **Enhances**: IL-10 (R-carvone) [329], IFNγ **Receptors/Channels**: In vitro, activate hTRPA1 with EC_50_ value at 112.2 µM, 81% [297] **Vapor pressure**: 0.115 mmHg at 25 °C	Caraway (*Carum carvi*) (S-carvone), spearmint (*Mentha spicata*) (R-carvone)	[275,297,328,329]
ß-Caryophyllene CID: 5281515 CAS: 87-44-5	Sesq MW: 204.35	**Suppresses**: IL-1ß, TNF-α, PGE2, iNOS, NO, ROS biomarkers, NF-kB, COX-2, IkBα; MMP8, Casp8, Casp4, IL-6 (in vivo, [16]), TREM1 signaling, VCAM-1 **Enhances**: IL-6 (in review, [278]), IL-19 Arg-1, urea, GSH parameters **Receptor**: CB2, (in vivo, [16])	Copaiba, lavender, rosemary, peppermint, common sage, clary sage, bushy lippia, Balkan heath	[16,278,319]
(+)-Catechin CID: 9064 CAS: 154-23-4	Flavonol MW: 290.27	**Suppresses**: in vitro, suppress LPS induced pro-inflammatory cytokines TNFα, IL-1 ß, IL-6.	Green tea, berries, grape seeds, kiwi, red wine, beer, cacao, etc.	[330]
Chamazulene CID: 10719 CAS: 529-05-5	Sesq MW: 184.28	**Suppresses**: TNFα, IL-6, MMP3, MMP9, p65 NF-kB, iNOS, COX2	chamomile *(Matricaria chamomilla*), wormwood (*Artemisia absinthium*), yarrow (*Achillea millefolium*)	[276,331]
1,8-Cineole CID: 2758 CAS: 470-82-6	Mono MW: 154.25	**Suppresses**: in vivo, LTB4, PGE2, TNF-α, IL-1ß, leukotriene B4, thromboxane B2, BALF, NO, IL-6, MMP-9, IL-4, IL-13, IL-17A in BALF, IL-5, MCP-1 in nasal lavage fluids, IFN-γ in lung tissues, NF-kB p65, JNK, TREM-1, NLRP3, p38, MKP-1 phosphatase, NLRP3 inflammasome activation, acetylcholinesterase activities **Enhances**: IL-10, IkBα; in vivo, wound healing by *Croton adamantinus* oil [332] **Receptors/Channels**: TRPM8 **Vapor pressure**: 1.90 mmHg at 25 °C	Eucalyptus, *Alpinia calcarata*(synonym: eucalyptol)	[275,278,296,300,301,302,303,332]
Cinnamaldehyde CID: 637511 CAS: 104-55-2	Phenyl MW: 132.16	**Suppresses**: VAM-1 and ICAM-1, NF-kB, NO, IL-1ß, IL-6, TNFα, iNOS, IRF3, COX2, PVGE2 (review [333]); in vivo, NO, IL-1ß, IL-18, TNF-α, IFN γ, HMGB-1 (high mobility group box 1 protein) [334] **Vapor pressure**: 2.89 × 10^−2^ mmHg at 25 °C	Cassia oil, cinnamon bark oil	[333,334]
(E)-Cinnamyl acetate CID: 5282110 CAS: 103-54-8	MW: 176.21	**Suppresses**: in vivo, iNOS, COX-2, NF-kB, IkBα, decreased paw edema after CARR administration **Increased**: activities of catalase, superoxide dismutase, glutathione peroxidase in paw tissue after Carr administration [335]; NO and PGE2 production (review [333])	Cinnamon (*Cinnamomum cassia*, *Cinnamomum osmophloeum*)	[333,335]
trans-Citral CID: 638011 CAS: 141-27-5	Mono MW: 152.23	**Suppresses**: NF-kB activation, COX-2, TRPV1-3, TRPM8, TRPV4, TRPA1 [284]; TNF, IL-6, IL-1ß, NO, macrophage activation, NLRP3 inflammasome activation; Compared to neral (isomer of cis-citral, neral), lower inhibitory effect on IL-1ß, iNOS, COX-2, and NLRP-2, and different inhibitory effects on phosphorylation of ERK1/2, JNK1/3, p38 and IkB [336] **Channels**: inhibitory effects on K_V_1.3 channel in vivo and in vitro [290], activate TRPA1, CB2 [284]	Lemongrass (*Cymbopogon citratus*); bushy lippia, lemon *myrtle*, *Litsea citrate*, *Litsea cubeba*, lemon tea-tree, *Ocimum gratissimum*, *Lindera citriodora*(synonym: geranial); 31.3% in *L. cubeba* fruits essential oil	[275,278,284,290,336,337,338]
cis-Citral CID: 643779 CAS: 106-26-3	Mono MW: 152.23	**Suppresses**: Compared to neral (isomer of citral, Citral B), lower inhibitory effect on IL-1ß, iNOS, COX-2, and NLRP-2, and different inhibitory effects on phosphorylation of ERK1/2, JNK1/3, p38 and IkB [336]	*Litsea cubeba*, 37.6% in *L. cubeba* fruits essential oil(synonym: neral)	[336]
Citronellol CID: 8842 CAS:106-22-9	Mono MW: 156.26	**Suppresses**: in vitro, down-regulated expression of ACE2 and TMPRSS2 [291] **Receptors/channels**: inhibitory effects on K_V_1.3 channel (ß-citronellol [290]) **Vapor pressure**: 0.02 mmHg at 25 °C	Geranium oil	[275,278,290,291]
Curcumin CID: 969516 CAS: 458-37-7	Polyp MW: 368.40	**Suppresses**: in vitro, suppressed phosphorylation of IKK ß and NF-kB p65 and suppressed degradation of IkBα [339] **Vapor pressure**: 3.08 × 10^−12^ mmHg at 25 °C	Turmeric (*Curcuma longa*)	[339,340,341]
Cyclocurcumin CID: 69879809 CAS: 153127-42-5	Polyp MW: 368.40	**Suppresses**: in vitro, higher neuroprotection than curcumin [342]	Turmeric (*Curcuma longa*); curcumin derivative	[341,342]
*p*-Cymene CID: 7463 CAS: 99-87-6	Mono MW: 134.22	**Suppresses**: in vivo, NO, NF-kB activity, TNFα, IL-1α, IL-10, suppressed licking behavior after formalin-injection **Vapor pressure**: 1.50 mmHg at 25 °C	Black cumin, rosemary, clove, Spanish oregano, valerian	[322,328]
Dehydrocostus lactone CID: 73174 CAS: 477-43-0	Sesq MW: 230.30	**Suppresses**: in vivo, NF-kB, COX2, TNF-α, IL-1ß, MCP-1, MPO, SOD, IL-6, IL-17, IL-23, IL-6/STAT3 inflammatory signaling pathway	Elecampane (*Inula helenium*), costus (*Saussurea lappa*)	[276,343]
Embelin CID: 3218 CAS: 550-24-3	benzoquinone MW: 294.4	**Suppresses**: in vivo, IP injection suppressed paw edema produced by carrageenan in rats	False black pepper (*Embella ribes*)	[344]
Epigallocatechin-3-gallate CID: 65064 CAS: 989-51-5	Polyphenol MW:458.4	**Suppresses**: review, alter NF-kB pathway, JAK/STAT pathway, PI3K/Akt pathway and suppress inflammation, down-regulate pro-inflammatory cytokines, COX, and reduce translocation of NF-kB to nucleus	Tea plant (*Camellia sinensis*)	[345]
Eugenol CID: 3314 CAS: 97-53-0	Mono MW: 164.20	**Suppresses**: in vivo, TNFα, IL-1ß, IL-6, NF-kB p65, suppress oxidative stress, reduced caspase-3 and p38 MAPK expressions in rats with spinal cord injury, IFNγ, IL-2, IL-10 **Enhances**: activate TRPA1, TRPV1 **Vapor pressure**: 0.0221 mmHg at 25 °C	Clove (*Eugenia caryophyllata*),	[296,346,347,348]
Eugenyl acetate CID: 7136 CAS: 93-28-7	Phenol MW: 206.24	**Suppresses**: in vitro, IFNγ, IL-2, IL-10	Clove (*Eugenia caryophyllata*), especially in bud, *Laurus nobilis*	[348]
Farnesol CID: 445070 CAS: 106-28-5	Sesq MW: 222.37	**Suppresses**: in vivo, slightly decreased IL-4, TNFα/IL-19 ratio **Enhances**: IL-10; contact allergen [349] **Vapor pressure**: 3.94 × 10^−5^ mmHg at 25 °C	Oils of lemongrass, chamomile, citronella	[276,349,350]
Ferruginol CID: 442027 CAS: 514-62-5	Diterp MW: 286.50	**Suppresses**: in vivo, TNFα, NF-kB, IL-1ß, COX2, MMP9, IL-6, iNOS in mice with ulcerative colitis	In needles of redwood (*Sequoia sempervirens*), heartwood of hinoki cypress (*Chamaecyparis obtusa)*	[351]
Geraniol CID: 637566 CAS: 106-24-1	Mono MW: 154.25	**Suppresses**: T cell proliferation, IL-2, TNF-α, IFN-γ; down-regulated expression of ACE2 and TMPRSS2 **Receptors/channels**: inhibitory to Kv1.3, in vivo, in vitro **Vapor pressure**: 3.0 × 10^−2^ mmHg at 25 °C	Geranium, Ylang-ylang, cinnamon, coriander, lemon grass, citronella grass, clary sage, roses	[275,278,290,291]
Geranyl acetate CID: 1549026 CAS: 105-87-3	Mono MW: 196.29	**Suppresses**: reduced nociceptive behavior **Receptors/Channels**: In vitro, activate hTRPA1 with EC50 value at 20.5 µM, 74% **Vapor pressure**: 3.3 × 10^−2^ mmHg at 25 °C	Citronella, lemongrass, neroli, geranium	[297,322]
6-Gingerol CID: 442793 CAS: 23513-14-6	Phenol MW:284.4	**Suppresses**: review, inhibition of pro-inflammatory cytokines, decreasing inducible NO synthase and TNFα by suppression of lkBα phosphorylation, NF-kB nuclear activation, and PKC α translocation, suppress IL-6, IL-8, SAA1 [352]; review, suppress IL-1 ß, IL-6, TNFα, down-regulate NF-kB/MAPK signaling pathway, iNOS, COX-2, suppress astrocyte overactivation, inhibit the expression of GFAP and TNFα in rat brain **Enhances**: Intercellular ROS, NO, iNOS, improved cognitive ability, improved memory	Ginger (*Zingiber officinale Roscoe*)	[352,353,354]
10-Gingerol CID: 168115 CAS: 23513-15-7	Phenol MW: 350.50	**Suppresses**: review, suppress IL-1 ß, IL-6, TNFα significantly; greatest anti-inflammatory and anti-oxidant effect compared to other gingerols [355]; review, suppress neuroinflammation [352]	Ginger (*Zingiber officinale Roscoe*)	[352,353,355]
18 α-Glycyrrhizin CID: 158471 18 ß-Glycyrrhizin CID: 3495 18 ß-Glycyrrhetinic acid CID:14982	Triterp	**Anti-inflammatory**: 18ß-Glycyrrhizin ,suppressed PGE2, ROS, TNFα, COX-2, iNOS, 18α-Glycyrrhizin, stronger anti-inflammatory effect than 18ß-Glycyrrhizin, 18ß-Glycyrrhetinic acid, anti-oxidant, decreased lipid peroxidation, suppressed NO, PGE2, ROS, iNOS, COX-2, LPS-induced TNFα, IL-6, IL-1ß [356]; 18ß-Glycyrrhetinic acid, suppressed LPS-induced iNOS, COX-2, TNFα, IL-6, IL-1ß [357]	Licorice *Glycyrrhiza uralensis* Fisch., *G. inflata* Bat., *G. glabra* L., roots and rhizomes	[356,357]
Herbacetin CID: 5280544 CAS: 527-95-7	Flavonol MW: 302.23	**Suppresses**: in vitro, RAW264.7 cells, reduced NO production, reduced the release of TNFα, IL-1ß, suppressed JNK kinase, NF-kB [358]; in vivo, in vitro, down-regulated MMP-9 and cathepsin K, significantly reduced LPS-induced inflammatory bone loss [359]	*Ephedrae herba*	[358,359]
Humulene CID: 5281520CAS: 6753-98-6	Sesq MW: 204.35	**Suppresses**: review, IL-5, CCL11, leukotriene B4 level, NF-kB and AP-1 activation (synonym: α-caryophyllene; α-humelene)	*Aniba parviflora*, cannabis, hop	[276,278,360]
Isoliquiritigenin CID: 638278CAS: 961-29-5	ChalconeMW: 256.25	**Suppresses**: in vitro, NO production, TNFα, IL-6, iNOS in IL-1ß treated cells	Licorice *Glycyrrhiza uralensis* Fisch., *G. inflata* Bat., *G. glabra* L., roots and rhizomes	[361]
(-)-Isopulegol CID: 170833 CAS: 89-79-2	Mono MW: 154.25	**Suppresses**: IL-1ß, TNFα, decreased albumin extravasation, leukocyte migration and myeloperoxidase (MPO) enzyme concentration **Receptor**: GABA_A_	Ironwort (*Sideritis*), chemical precursor to menthol, also found in lemongrass and geranium	[285,362]
Kahweol CID: 114778 CAS: 6894-43-5	Diter MW: 314.40	**Suppresses**: review, PGE2, NO synthesis, COX2, iNOS, inhibit activation of inhibitor kB kinase **Enhances**: Nrf2/HO-1 pathway, increase the expression of HO-1	Coffee beans	[321]
Kaempferol CID: 5280863 CAS: 520-18-3	Flavonol MW: 286.24	**Suppresses**: in vitro, NOD-like receptor family pyrin domain containing 3 (NLRP3) inflammasome action **Vapor pressure**: 0.0 ± 1.5 mmHg (25 °C)	Grapes, tomatoes, broccoli	[315]
Kazinol J CID: 21637732	Polyphenol MW:410.5	**Activates**: in vivo, in vitro, suppress pro-inflammatory responses by activating AMPK in 3T3-L1 adipocyte	paper mulberry (*Broussonetia papyrifera*)	[320]
Kirenol CID: 15736732	Diter MW: 338.50	**Suppresses**: in vitro, in vivo, IL-6, IL-8, MMP-9, MAPK, p65, P50, JAK gene expression in RA-FLS (rheumatoid arthritis-associated synovial fibroblasts)	*Siegesbeckiae Herba* (*S. pubescens* Makino *S. orientalis* L., *S. glabrescens* Makino)	[363]
Licochalcone A CID: 5318998 Licochalcone B CID: 5318999 Licochalcone C CID: 9840805 Licochalcone D CID: 10473311 Licochalcone E CID: 46209991	Flavonoid	**Suppresses**: key factors for biological activities, Licochalcone A, suppress NO, IL-6, PGE2 IL-4, IL-5, IL-13, Licochalcone C, suppressed iNOS, Licochalcone E suppressed PKC/JNK, ERK1/2, iNOS, COX-2, IL-6, IL-1ß, IL-12 p40, TNF- α, AKT, p38 MAPK	Licorice *Glycyrrhiza uralensis* Fisch., *G. inflata* Bat., *G. glabra* L.	[356]
D-Limonene CID: 440917 CAS: 5989-27-5	Mono	**Suppresses**: TNF-α, IL-1ß, IL-6, NF-kB, COX-2, iNOS, NO levels, p38, JNK activation; lipoxygenase; in vitro, down-regulated expression of ACE2 and TMPRSS2 [291] **Receptors/Channels**: In vitro, activate hTRPA1 with EC_50_ value at 54.3 µM, 83% [297] **Vapor pressure**: 1.98 mmHg at 25 °C	*Citrus aurantium* (bitter orange, daidai)	[275,278,291,297]
Linalool CID: 6549 CAS: 78-70-6	Mono MW: 154.25	**Suppresses**: TNF-α, IL-6, NO, IL-1ß, PGE2, p38, MAPK, NOS2, COX2, IL-18, IFN-γ, HMGB-1, MLNs, Nrf2 markers, iNOS expression, NF-kB activation, JNK activation, phosphorylation of IkBα protein, p38, c-JNK, ERK; in vivo, NO, IL-1ß, IL-18, TNF-α, IFN γ, HMGB-1 (high mobility group box 1 protein) [334] **Enhancees**: Nuclear Nrf-2 protein translocation; anxiolytic effect through GABA [19]; enhance recovery after ischemia [21] **Receptors, pathways involved**: GABA, TRPA1, TRPM8; inhibitory effects on K_V_1.3 channel in vivo and in vitro [290]; In vitro, activate hTRPA1 with EC_50_ value at 167.7 µM, 89% [297] **Vapor pressure**: 0.159 mmHg at 23.5 °C	Mint, rosewood, lavender, laurel, sweet basil, *Cinnamomum osmophloeum* Kanehira	[19,21,275,278,290,296,297,334]
Linalyl acetate CID: 8294 CAS: 115-95-7	Mono MW: 196.29	**Suppresses**: In vitro, suppressed TNFα induced E-selection, P-selection, vascular cell adhesion molecule-1 (VCAM1), suppressed NF-kB activation [364] **Receptors/Channels**: In vitro, activate hTRPA1 with EC_50_ value at 30.2 µM, 69% [297] **Vapor pressure**: 0.111 mmHg at 25 °C	Bergamot, lavender; acetate ester of linalool	[275,297,364]
Luteolin CID: 5280445CAS: 491-70-3	polyphenol MW: 286.24	**Suppresses**: review, in vivo, in vitro, regulates cytokines by suppressing IL-1ß, IL-6, IL-2, IL-8, IL-12, IL-17, TNFα, which are pro-inflammatory cytokines, and enhancing IL-10, which is anti-inflammatory cytokine	apples, carrots, celery, olive oil, rosemary, thyme, oregano, chamomile and many others	[365]
1-Menthol CID: 16666 CAS: 89-78-1	Mono MW: 156.26	**Suppresses**: in vivo, increased survival rates in mice with myocardial infarction. Suppressed TNFα, IL-1ß, IL-6, monocyte chemoattractant protein 1 (MCP-1) [366]; in vivo and in vitro, inhibits acid-induced inflammation, suppress TNFα, IL-1ß, IL-6 through regulating TRPV1 [367] **Receptors/Channels**: TRPM8, TRPV1	Genus *Mentha*, Corn mint, peppermint	[275,300,337,366,367]
Menthone CID: 26447 CAS: 14073-97-3	Mono MW: 154.25	**Suppresses**: in vivo, alleviate depression symptoms, suppressed expression of pro-inflammatory cytokines, IL-1ß, IL-6, TNFα, NLRP3 inflammasome [368]	Genus *Mentha*	[275,368]
Methyl eugenol CID: 7127 CAS: 93-15-2	Phenyl MW: 178.23	**Suppresses**: in vitro, inhibited the release of ß-hexosaminidase, TNFα IL-4, PGE2, prostaglandin D2, leukotriene B4, leukotrience C4, Syk phosphorylation and expression ERK1/2, p38, JNK phosphorylation, cytosolic phospholipase A2, 5-lipoxygenase phosphorylation, COX2 expression, considered to inhibit allergic response by these suppressions **Enhances**: in vivo, wound healing by *Croton adamantinus* oil [332] **Receptors/channels**: Na_v_1.7, GABA_A_ **Vapor pressure**: 0.012 mmHg at 25 °C	*Croton adamantinus* (major chemical compound methyl eugenol and 1,8-cineole) [332]; Anti-allergic, antinaphylactic, antinociceptive, anti-inflammatory effects	[18,287,332,369]
Myrcene CID: 31253 CAS: 123-35-3	Mono MW: 136.23	**Suppresses**: review, NO, iNOS, NF-kB, p38, JNK activation **Vapor pressure**: 2.09 mmHg at 25 °C	Dill, cinnamon, coriander, lemon grass, citronella grass, English lavender, bushy lippia, common sage, clary sage, myrcia, bay, rosemary, cannabis, ylang-ylang, wild thyme, parsley, cardamom, hops	[275,278,337]
Nootkatone CID: 1268142 CAS: 4674-50-4	Sesq MW: 218.33	**Suppresses**: in vivo, suppressed edema, inhibition of IL-1ß, TNFα production, inhibition of COX-2 activity, anti-H1 receptor [370]; in vitro, show synergistic effect of suppressing inflammation with schisandrin, a polyphenol included in *Schisandra* genus [371] **Vapor pressure**: 0.003 mmHg at 25 °C	In many species of *Citrus*, black cardamom (*Alpinia oxyphylla*)	[276,370,371]
(E)-ß-Ocimene CID: 5281553 CAS: 3779-61-1	Mono MW: 136.23	**Suppresses**: NO production inhibition; NO scavenging effect; inhibited inducible NO synthase expression	Basil (*Ocimum basilicum*), water hemlock (*Ocenanthe crocata)* common wormwood or absinthe (*Artemisia absinthium*) and many others	[17,337,372]
Oleanolic acid CID: 10494 CAS: 508-02-1	Tri MW: 456.70	**Suppresses**: IL-6 and TNF-α [373]; in vitro, inhibit NF-kB activation [374] **Enhances**: cell viability and release of lactate dehydrogenase	Olive (*Oleaceae*), grapes (*Vitis vinifera*)	[373,374]
Osthole CID: 10228 CAS: 484-12-8	Coum MW: 224.28	**Suppresses**: in vivo and in vitro, NO, PGE2, TNFα, IL-6, iNOS, COX-2, p38 MAPK, IkB [375] **Receptors/Channels**: In vitro, activate hTRPA1 with EC_50_ value at 6.0 µM, 92% [297] **Vapor pressure**: 6.9 × 10^−6^ mmHg at 25 °C	Cnidium (*Cnidium monnieri*), shishiudo or du huo (*Angelica pubescens*)	[297,375,376]
Parthenolide CID: 7251185 CAS: 20554-84-1	Sesq MW: 248.32	**Suppresses**: Binds directly to IkB kinase ß (IKKß) and inhibits its activity. IkB is an inhibitor of NF-kB and becomes phosphorylated by IkB kinase complex, IKK. There are two forms: IKKα and IKKß. (in vitro, [377]), thus parthenolide suppress NF-kB by targeting IkB kinase (in vitro, [378]); Parthenolide depleted feverfew still has anti-inflammatory effects (in vitro, [379]) **Others**: allergen (human subjects, [380])	Feverfew (*Tanacetum parthenium*)	[337,377,378,379,380]
Perillyl alcohol CID: 10819 CAS: 536-59-4	Mono MW: 152.23	**Suppresses**: review, oxidative stress and lipid peroxidation, IL-1ß, TNFα, IL-6, COX-2, NOS-2, NF-kB **Enhances**: levels of glutathione, catalase, glutathione peroxidase, and glutathione reductase **Others**: D-limonene metabolite; strong candidate for cancer treatments; induce apoptosis to cancer cells; oral treatment cause intestinal side effects; tissue regeneration improved; blocked formalin-, capsaicin-, and glutamate-induced nociceptive behavior	Lavender, sage, peppermint, lemongrass, cannabis, hop	[275,360]
α-pinene CID: 2723720	Mono MW: 136.23	**Suppresses**: review, NF-kB, ERK, JNK; G2/M-phase cell cycle arrest miR-221 expression level **Enhances**: CDKN1B/p2-CDK1 and ATM-p52- Chk2 pathways activated	Pine, coniferous species, sagebrush, ironwort, sage, *Cannabis*, *Humulus*	[278,337]
(R)-(+)-Pulegone CID: 442495 CAS: 89-82-7	Mono MW: 152.23	**Suppresses**: in vivo, suppressed skin thickness and scratching, serum IgE level, IL-4, IFN-γ, IL-6, TNF-α, IL-1ß, phosphorylation of MAPK, inhibited IkBα degradation and NF-kB activation [381]	Mint species, for example, *Mentha* spicata *Mentha pulegium*, *Mentha piperita*, *Hedeoma multiflorum*, *Minthostachys mollis*, *Satureja boliviana*, *Satureja odora*,	[275,381,382]
Sabinene CID: 18818 CAS: 3387-41-5	Mono MW: 136.23	**Suppresses**: in vitro, using whole essential oil from hallabong flower which included 34.75% sabinene, suppressed NO, PGE2, COX-2, TNF-α, IL-6, IL-1 ß [383]; suppressed NO production in lipopolysaccharide and IFN-γ stimulated macrophages [372]	*Oenanthe crocata*, 34.75% in Hallabong flower (*Citrus unshiu* Marcov × *Citrus sinensis* Osbeck) × *Citrus reticulata* Blanco	[372,383]
Santamarine CID: 188297 CAS: 4290-13-5	Sesq MW: 248.32	**Suppresses**: Suppress NF-kB activation, induces oxidative stress in cancer cells [384]	Southern magnolia (*Magnolia grandiflora*), weakleaf bur ragweed (*Ambrosia confertiflora*)	[275,384]
6-shogaol CID: 5281794 CAS: 555-66-8	MW: 276.40	**Suppresses**: review, inhibits direct binding between intercellular adhesion molecule, inhibits production of prostaglandin E2 and pro-inflammatory cytokines, together with 10-gingerol, suppressed NO, IL-1 ß, IL-6, TNF-α	Ginger (*Zingiber officinale*); Dehydration product of gingerol	[352]
Spathulenol CID: 92231 CAS: 6750-60-3	Sesq MW: 220.35	**Suppresses**: in vivo and in vitro. Using whole essential oil which contains 80% spathulenol. Inflammation measured by swelling in carrageenan-induced paw oedema	Brazilian guava (*Psidium guineense Sw*.)	[385]
α-terpinene CID: 7462 CAS: 99-86-5	Mono MW: 136.23	**Suppresses**: review, COX21 **Enhances**: review, Strong anti-oxidant; increased longevity of mice infected with *Trypanosoma evansi*	Tea tree (*Melaleuca alternifolia*), *Litsea*, cannabis, hops	[275,360]
γ-terpinene CID: 7461 CAS: 99-85-4	Mono MW: 136.23	**Suppresses**: review, TNF-α, IL-1ß, IL-6 **Enhances**: review, IL-10, COX-2, PGE2; *In vivo*, anti-nociceptive effect to formalin, capsaicin, glutamate-induced pain in rats, cholinergic and opioid systems were involved in anti-nociceptive effects [386] **Vapor pressure**: 1.09 mmHg	Narrow-leaved paperbark (*Melaleuca alternifolia*), thyme, savories (*Satureja*), cannabis, hops	[278,360,386]
Terpinen-4-ol CID: 11230CAS: 562-74-3	MonoMW: 154.25	**Suppresses**: NF-kB, NLRP3, IkBα, NF-kB p65; IL-1ß, IL-6, IL-10 **Enhances**: PPAR-γ, **Route**: GABAergic system **Vapor pressure**: 0.04 mmHg at 25 °C	Tea tree (*Melaleuca alternifolia*), lavender	[275,278]
α-terpineol CID: 17100 CAS: 98-55-5	Mono MW: 154.25	**Suppresses**: Nitrite production, NF-kB, IL-1ß, IL1R1; IL-1ß, IL-6, IL-10, IL-4, IL-17 TNF-α, COX-2, iNOS, **Enhances**: IL-10 **Vapor pressure**: 0.0423 mmHg at 24 °C	*Melaleuca* genus, eucalyptus, Balkan heath (*Erica spiculifolia*), cajuput, pine, orange juice	[275,278,319,387,388]
Terpinolene CID: 11463 CAS: 586-62-9	Mono MW: 136.23	**Suppresses**: IL-6, TNF-α, NO **Vapor pressure**: 0.74 mmHg at 25 °C	*Melaleuca* genus; Myrtle (*Myrtus communis* L., Myrtaceae)	[278]
Thymol CID: 6989 CAS: 89-83-8	Mono MW: 150.22	**Suppresses**: in vitro, IL-8, TNFα, reactive oxygen species (ROS) [389] **Enhances**: barrier function **Vapor pressure**: 0.016 mmHg at 25 °C	*Lippia gracilis* Schauer, oregano species, thyme species, Balkan heath (*Erica spiculifolia*)	[275,319,337,389]
Thymoquinone CID: 10281 CAS: 490-91-5	Mono MW: 164.20	**Suppresses**: in vivo and in vitro, suppressed NO, iNOS, TNFα, COX2, IL-6, IL-1ß in lipopolysaccharide-stimulated murine macrophage-like RAW264.7 cells, suppression of IRAK-linked AP-1/NF-kB pathways, suppressed hepatitis and gastritis symptoms in mouse models [390]	Black seed (*Nigella sativa*)	[275,390]
Tomentosin CID: 155173 CAS: 33649-15-9	Sesq MW: 248.32	**Suppresses**: in vitro, NF-kB, MAP, NO, PGE2, iNOS, COX-2, TNF-α, IL-6, p65	*Inula japonica*, *Inula viscosa* (syn. *Dittrichia viscosa Greuter*),	[276,391]
Tussilagone CID: 13919185 CAS: 104012-37-5	Sesq MW: 390.50	**Suppresses**: in vitro, suppressed production of NO, TNF-α, PGE2, iNOS, COX2 [392]; in vivo, protective effect against dextran sulfate sodium-induced acute colitis in mice, TNF-α, IL-6, and myeloperoxidase activity reduced [393] **Enhances**: heme oxygenase-1	Flower and buds of *Tussilago farfara*	[276,392,393]
Ursolic acid CID: 64945 CAS: 77-52-1	Tri MW: 456.70	**Suppresses**: in vitro, IL-6 and TNF-α [373]; in vitro, inhibit NF-kB activation [374] **Enhances**: cell viability and release of lactate dehydrogenase **Vapor pressure**: 3.49 × 10^−14^ mmHg at 25 °C	Various fruits and vegetables (apples, berries, peppermint, lavender, oregano, etc.)	[373,374,394]
Valencene CID: 9855795 CAS: 4630-07-3	Sesq MW: 204.35	**Suppresses**: in vivo and in vitro, IL-1ß, IL-6, IL-13, NF-kB, CXCL8, GM-CSF, I-CAM, reduced atopic dermatitis-like symptoms [395] **Enhances**: skin barrier protein, involucrin increased in murine skin [395]	Nut grass (*Cyperus rotundus*), Citrus; orange peel oil	[276,395]

Mono: monoterpene (number of C atoms C_10_, 2 isoprene units), Sesq: sesquiterpene (C_15_, 3 isoprene units), Diterp: diterpene (C_20_, 4 isoprene units), Tri: triterpenoid, Guaia: guaiacol, Mero: a chemical compound containing terpenoid structure, NO: nitric oxide, COX2: cyclooxygenase 2, iNOS: nitric oxide synthase, TNFα: tumor necrosis factor α, NF-kB: nuclear factor-kappa B, PPAR-α: peroxisome proliferator activated receptor α, TRP: transient receptor potential, -: none or not so many studies, or no in vitro and/or in vivo studies at chemical compound level to be convincing.

### 7.4. Terpenes and Other Volatile Phytochemicals with Anti-Viral Effects

There have been studies on the possible use of natural products to treat or prevent infections by various virus species (for review [396,397,398,399]). For example, studies have shown the anti-viral effects of carvacrol on murine norovirus [400], the effects of α-terpinene, γ-terpinene, α-pinene, p-cymene, terpinene-4-ol, α-terpineol, thymol, citral, and 1,8-cineole on herpes simplex virus type 1 (HSV-1) [401,402] and respiratory syncytial virus (RSV) [403]. As SARS-CoV-2 belongs to the same coronavirus family as SARS-CoV and MERS-CoV, sharing almost 80% and 50% genomic homology with them, respectively [402], it is reasonable to focus on the results of the studies on SARS-CoV and MERS-CoV as well as in silico studies on SARS-CoV-2.

Various phytochemicals are found to have anti-viral activity on SARS-CoV and MERS-CoV. These studies have found that, depending on the chemical compounds, the mechanisms of action are different. Betulinic acid, a triterpene included in the bark of Downy birch (*Betula* *pubescens*), was found to inhibit the activity of 3-chymotrypsin-like protease (3CL^pro^) (3CL^pro^: 3-chymotrypsin-like protease or main protease or main protease (M^Pro^) is one of the proproteins that SARS-CoV and SARS-CoV-2 produces. 3CL^pro^ (11 cleavage sites) and papain-like protease (PL^pro^) (3 cleavage sites) process the polyproteins translated from the viral RNA. 3CL^pro^ processes its own *N*- and *C*- terminal auto-processing sites as well [404]. 3CL^pro^ and PL^pro^ have essential roles in the replication of the virus. Compounds that have binding affinity with these proteases are considered to be promising candidates to become drugs to treat coronavirus induced diseases because of their essential roles in the replication and also because there are no human proteases with similar cleavage specificity [405,406]) of the SARS-CoV virus [397,407], which is crucial for its replication. In silico analyses have shown that betulinic acid fits in the 3CL^pro^ substrate-binding pocket of SARS-CoV [407]. Other studies have shown affinity to main protease 5R7Y, affinity to the S glycoprotein, inhibitory effects on viral growth, inhibition of the ACE2 receptor, inhibition of papain-like protease (PL^pro^), and binding affinity to other proteases [397].

**Figure 9:**
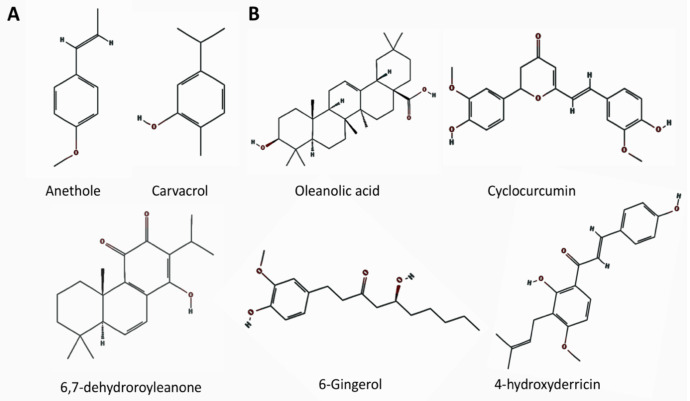
Chemical compounds with binding affinity to SARS-CoV-1 or 2. (**A**) Chemical compounds with binding affinity to the S glycoprotein. (**B**) Chemical compounds with binding affinity to M^pro^ and/or 3CL^pro^.

Following the outbreak of SARS-CoV-2 in December 2019, there have been in silico studies published exploring the possibility of utilizing phytochemicals to treat or prevent COVID-19. Studies on binding affinity suggested that there are good possibilities of using phytochemicals and the mechanisms of action would be different because of differences in the type of molecular structures that they showed affinity. For example, anethole, cinnamaldehyde, carvacrol, geraniol, cinnamyl acetate, 4-terpineol, thymol, pulegone, and menthol are found to have binding affinity to the receptor binding domain (RBD) of the S glycoprotein of SARS-CoV-2 (Figure 9), and binding of these terpenes to the virus is expected to disturb the virus from binding to host cells and delay/block the infection [408] (Table 3). Gingerol, which is a phenolic compound in ginger (*Zingiber officinale*), also has high affinity to a SARS-CoV-2 main protease 5R7Y (−15.7591 kJ/mol) [409] (Figure 9). It also shows binding affinities to RNA binding proteins 6W4B (-11.4082 kJ/mol) and 6VSB (−12.9523 kJ/mol), and to S glycoprotein 6M3M (−12.8835 kJ/mol) [409], which will affect the replication of the virus and entering the host cells.

Importantly, there are flavonoids that produce opposite effects, which were found using influenza virus [410], i.e., some flavonoids inhibited viral replication whereas some enhanced it. The differences between these two opposite influences were found to be based on the differential influences of the flavonoids on the mitogen-activated protein (MAP) kinase pathways [410]. The flavonoids that had an anti-influenza virus effect, for example, hesperidin, up-regulated p38 and JUN N-terminal kinase (JNK) activation and down-regulated extracellular-signal-regulated kinase (ERK), and those that enhanced the replication of the influenza virus, kaempferol, down-regulated p38 and JNK and up-regulated ERK [410]. These studies suggest that the influences of flavonoids on cell-autonomous immunity through modulating MAP kinase pathways could be one of the key aspects that needs to be addressed in selecting the flavonoids to be used. Another factor that may have importance is the general assumption that prenylation of flavonoids may enhance their bioactive level by changing affinity to the target [411]. Chemically modifying them to make them hydrophobic may thus enhance their anti-viral effects.

**Table 3:** Phytochemicals with binding affinity to SARS-CoV-1 or 2 or shown to have anti-viral effects in in vitro assays.

Chemical Compounds	Type	Targeted Virus, Parts of the Virus, and Effects	Essential Oils and Herbs with the Chemical Compoundsas Major Chemical Constituents	References
Acetoside CID: 5281800 CAS: 61276-17-3	Phenol MW: 624.60	Covalent docking with 3CL^pro^ (6LU7) of COV2 and XP docking and covalent docking with spike RBD (6M0J) of S glycoprotein	Many plants, example, *Ligustrum purpurascens*, *Rehmannia glutinosa*	[412]
Andrographolide CAS: 5508-58-7 CID: 5318517	Diter MW: 350.4	**Suppress**: suppress the 3CL^pro^ activities of SARS-CoV-2;	Green chiretta (*Andrographis paniculate*)	[312,341]
Anethole CID: 637563 CAS: 104-46-1	Mono MW: 148.20	COV2 by binding affinity to RBD of S glycoprotein	Star anise (*Illicium verum)*, *Apiaceae* (fennel, celery, carrot, parsley), *Myrtaceae* (myrtle, bay rum tree, clove, guava)	[408]
Betulonic acid CID: 122844 CAS: 4481-62-3	Triterp MW: 454.70	COV1, HCPE at >10 µM 3CL^pro^, EC_50_ 0.63 µM, CC_50_ 112 µM, Selectivity 180	Formosan juniper (*Juniperus formosana*)	[407]
Betulinic acid CID: 64971 CAS: 472-15-1	Triterp MW: 456.70	COV1, HCPE at >3.3 µM, EC_50_ >10 µM, CC_50_ 150 µM, Selectivity <15	Downy birch or white birch (*Betula pubescens*)	[407]
(-)-α-cadinol CID: 6431302	Sesq MW: 222.37	COV1, HCPE at >1 uM, EC_50_ 4.44 µM, CC_50_ 76.8 µM, Selectivity 17.3	Hinoki cypress (*Chamaecyparis obtuse* var. *formosana*, or *Chamaecyparis taiwanensis*)	[407]
Cafestol CID: 108052 CAS: 469-83-0	Diter MW: 316.40	In silico, COV2, PL^pro^, Guanine-N7 methyl transferase (ExoN)	Coffee beans	[413]
Carvacrol CID: 10364 CAS: 499-75-2	Mono MW: 150.22	COV2 by binding affinity to RBD of S glycoprotein [408]; COV2 by binding affinity to M^pro^ [414]	Oregano (*Origanum* species), thyme (*Thymus vulgaris*), Spanish origanum (*Thymus capitatus*), pepperwort (*Lepidium flavum*), black cumin (*Nigella sativa*), summer savory (*Satureja hortensis*), winter savory (*Satureja montana*)	[408,414]
Cinnamaldehyde CID: 637511 CAS: 104-55-2	Phenyl MW: 132.16	COV2 by binding affinity to RBD of S glycoprotein	Cinnamon (*Cinnamomum zeylanicum*)	[408]
(E)-Cinnamyl acetate CID: 5282110 CAS: 103-54-8	Ester MW: 176.21	COV2 by binding affinity to RBD of S glycoprotein	Cinnamon (*Cinnamomum zeylanicum*)	[408]
Curcumin CID: 969516 CAS: 458-37-7	Polyp MW: 368.40	In silico, Covalent docking with 3CL^pro^ (6LU7, 5R82) of COV2; in silico, COV2, 3CL^pro^	Turmeric (*Curcuma longa*)	[341,412,413]
Cyclocurcumin CID: 69879809 CAS: 153127-42-5	Polyp MW: 368.4	In silico, 3CL^pro^ (5R82; G score -6.77)	Turmeric (*Curcuma longa*)	[341]
Dehydroabieta-7-one CID: 11289118	Diterp MW: 284.40	COV1, HCPE at >10 µM, EC_50_ 4 µM, CC_50_ 305.1 µM, Selectivity 76.3	Hinoki cypress (*Chamaecyparis obtuse* var. *formosana*, or *Chamaecyparis taiwanensis*)	[407]
6,7-dehydroroyleanone CID: 2751794 CAS: 6855-99-8	Diterp MW: 314.40	COV1, HCPE at >10 µM, EC_50_ 5.55 µM, CC_50_ 89.7 µM, Selectivity 16.2	Hinoki cypress (*Chamaecyparis obtuse* var. *formosana*, or *Chamaecyparis taiwanensis*)	[407]
3ß,12-diacetoxyabieta-6,8,11,13-tetraene Not in pubchem	Diterp	COV1, HCPE at >3.3 µM, EC_50_ 1.57 µM, CC_50_ 303.3 µM, Selectivity 193	Formosan juniper (*Juniperus formosana*)	[407]
Embelin CID: 3218 CAS: 550-24-3	Quinone MW: 294.40	In silico, COV2, binding affinity to Nst7-Nsp8 complex [413]; with 3CL^pro^	False black pepper (*Embella ribes*), dotted loosestrife (*Lysimachia punctata*)	[413,415]
(-)-Epicatechin CID: 72276 CAS: 490-46-0	Flavonol MW: 290.27	In silico, COV2, binding affinity to PLpro, 3Lpro, hACE2, Nsp7-Nsp8, Guanine-N7 methyl transferase (ExoN), RdRp, Helicase, NendoU, M, NC	Tea plant (*Camellia sinensis*)	[413]
Epigallocatechin-3-gallate CID: 65064 CAS: 989-51-5	Polyphenol MW: 458.4	in vitro, COV2, suppress 3CL^pro^	Tea plant (*Camellia sinensis*)	[416]
Ferruginol CID: 442027 CAS: 514-62-5	Diterp MW: 286.50	COV1, HCPE at >3.3 µM, EC_50_ 1.39 µM, CC_50_ 80.4 µM, Selectivity 58	Hinoki cypress (*Chamaecyparis obtuse* var. *formosana*, or *Chamaecyparis taiwanensis*)	[407]
Forskolin CID: 47936 CAS: 66428-89-5	Diterp MW: 410.5	COV1, HCPE at >3.3 µM, EC_50_ 7.5 µM, CC_50_ 674 µM, Selectivity 89.8	*Coleus barbatus*	[407]
Geraniol CID: 637566 CAS: 106-24-1	Mono MW: 154.25	COV2 by binding affinity to RBD of S glycoprotein	*Cymbopogon* (palmarosa, citronella, lemongrass), *Lavendula* (lavender), rose	[408]
Gingerol CID: 442793 CAS: 23513-14-6	Phenol MW: 284.4	COV2 by binding affinity to 3CL^pro^ (5R7Y), to RNA binding proteins 6W4B and 6VSB, and to S glycoprotein (6M3M)	Ginger	[409]
Glycyrrhizin (glycyrrhizic acid) CID: 14982 CAS: 1405-86-3	Triterp MW: 822.9	COV1, EC_50_ 300 mg/L, CC_50_ >20,000 mg/L, Selectivity >67 if added during and after virus absorption	Liquorice (*Glycyrrhiza glabra*)	[417]
		COV1, EC_50_ 365 µM, CC_50_ >24,000 µM, Selectivity >65	Liquorice (*Glycyrrhiza glabra*)	[418]
Herbacetin CID: 5280544 CAS: 527-95-7	Flavonol MW: 302.23	COV1 suppress 3CL^pro^	*Ephedrae herba*	[411,419]
ß-hydroxyabieta-9(11),13-dien-12-one Not in pubchem	Diterp	COV1, HCPE at >3.3 µM, EC_50_ 1.47 µM, CC_50_ >750 µM, Selectivity >510	Hinoki cypress (*Chamaecyparis obtuse* var. *formosana*, or *Chamaecyparis taiwanensis*)	[407]
4-hydroxyderricin CID: 6438503 CAS: 55912-03-3	Chalcone MW: 338.4	COV1; suppress 3CL^pro^, PL^pro^	Ashitaba (*Angelica keiskei*)	[411]
7ß-hydroxydeoxycryptojaponol Not in Pubchem	Diterp	COV1, HCPE at >10 µM, EC_50_ 1.15 µM, CC_50_ 127 µM Selectivity 111	Japanese cedar or sugi (*Cryptomeria japonica*)	[407]
Kaempferol CID: 5280863 CAS: 520-18-3	Flavonol MW: 286.24,	COV2, 3CL^pro^ Vapor Pressure: 0.0 ± 1.5 mmHg at 25 °C	Grapes, tomatoes, broccoli; Green chiretta (*Andrographis paniculata* (Burm. f))	[341]
Kazinol J CID: 21637732	Polyphl MW:410.5	In silico, COV2, binding affinity with 3CL^pro^	Paper mulberry (*Broussonetia papyrifera*)	[420]
Luteolin CID: 5280445CAS: 491-70-3	Flavonone MW: 286.24	COV2 by binding affinity to 3CL^pro^ [421]; to 3pro and ACE2 [422]	Dyer’s weed (*Reseda luteola*)	[415,421,422]
1-Menthol CID: 16666 CAS: 89-78-1	Mono MW: 156.26	COV2 by binding affinity to RBD of S glycoprotein	Peppermint oil	[408]
Murrayanine CID: 96942 CAS: 723-97-7	Carbazole MW: 225.24	In silico, COV2, inhibitory to Nsp10-Nsp16 complex	Curry tree (*Murraya koenigii*)	[413]
Murrayaquinone-A CID: 127481 CAS: 100108-66-5	Carbazole MW: 211.22	In silico, COV2, inhibitor of Nsp9, for H-bond with Val110		[413]
Oleanolic acid CID: 10494 CAS: 508-02-1	Triterp MW: 456.70	COV2 by binding affinity to 3CL^pro^	Olive (*Oleaceae*), grapes (*Vitis vinifera*)	[414]
Pectolinarin CID: 168849 CAS: 28978-02-1	Flavone MW: 622.6	COV1 suppress 3CL^pro^		[419]
Pinusolidic acid CID: 25880646	Diterp MW: 332.4	COV1, HCPE at >10µM, EC_50_ 4.71 µM, CC_50_ > 750 µM, Selectivity > 159	Hinoki cypress (*Chamaecyparis obtuse* var. *formosana*, or *Chamaecyparis taiwanensis*)	[407]
(+)-Pulegone CID: 442495 CAS: 89-82-7	Mono MW: 152.23	COV2 by binding affinity to RBD of S glycoprotein	Creeping charlie (*Glechoma hederacea* or *Nepeta Glechoma*), catnip (*Nepeta cataria*), Pennyroyal (*Mentha pulegium*)	[408]
Quercetin CID: 5280343 CAS: 117-39-5	Flavonole MW: 302.23	COV1 suppress 3CL^pro^		[411,423]
Rhoifolin	Flavone	COV1 suppress 3CL^pro^		[419]
Rutin	Rutin	COV2 by binding affinity to RBD (6M0J) of S glycoprotein and to 3CL^pro^ (6LU7)		[412,423]
Solanine	alkaloid	COV2 by binding affinity to RBD (6M0J) of S glycoprotein and to 3CL^pro^ (6LU7)		[412]
4-Terpineol	Mono	COV2 by binding affinity to RBD of S glycoprotein	Major chemical constituent of Tea tree oil, lavender, turpentine oils	[408]
Thymol CID: 6989 CAS: 89-83-8	Mono MW: 150.22	COV2 by binding affinity to RBD of S glycoprotein; HSV-1	Isomeric with carvacrol; Thyme (*Thymus vulgaris*)	[408] (COV2); [424] (HSV)
Ursolic acid CID: 64945 CAS: 77-52-1	Triterp MW: 456.70	COV2 by binding affinity to 3CL^pro^	Peels of fruits	[414]
Xanthoangelol		COV1 suppress PL^pro^ [425], 3CL^pro^ [411]	Ashitaba (*Angelica keiskei*)	[425]

Sesq: sesquiterpene, Diterp: diterpene, Pheno: phenolic glycoside, Phenyl: phenylpropanoid, Flav: flavonoid, Polyp: polyphenol, Triterp: triterpenoid, COV2: SARS-CoV-2, COV1: SARS-CoV, HCPE: high cytopathogenic effect, Selectivity: CC_50_ (µM)/EC_50_(µM) where CC_50_ is cytotoxic concentration which reduced the cell viability to 50% and EC_50_ is effective concentration for the inhibition of viral replication to 50% of control [407].

Table 4 shows some examples of the herbs and essential oils described so far, which include chemical constituents with anti-inflammatory effects and that have binding affinity to SARS-CoV-2. They are the candidate essential oils for a new smell training combination from the perspective of the bioactive properties of their chemical constituents on anti-inflammation and binding affinity to SARS-CoV-2. These are volatile chemical compounds and it is possible to use them in smell training as well as in taste training.

In this table, examples of the percentage of the chemical compounds included in parts of the plants and oils are also indicated. It is important in relation to utilizing the essential oils of these herbal plants that there are large differences in the concentration of the chemical compounds. Various factors affect the concentration included, i.e., the geographical location, the season, the parts of the plant used, old leaves compared to young leaves, and even the weather of the year when harvested (see Koyama and Heinbockel 2020 [17] for review). It is thus important to obtain the chemical constituent profile of the essential oils when they are used in smell training, and, if possible, compensate the amount if some chemical compounds are at lower concentration in order to control the conditions. This is one of the facts that is less recognized and would possibly affect the results significantly. That is, many products are on the market with the same name, for example, essential oils of lavender, without the precise chemical profile available. When essential oils are used in smell training, it will be important to control the concentrations of the chemical constituents in order to know what is used and to determine what chemical compound profile produces the best results reliably. The list in Table 4 can be used as a list of candidates to test their effects but it is also necessary to test at several different concentrations of the major constituents to determine what concentration and combination of the chemical constituents brings the best results in treating chemosensory dysfunction. Taking into consideration the studies so far (for example [13,15]) and taking into consideration the list in Table 4, there are possibilities for developing a new essential oil with the chemical constituents at the concentrations that bring the most reliable effects for facilitating the recovery of chemical senses. Basic studies on the effects of the phytochemicals and their concentrations are needed to reach this goal.

**Table 4:** Essential oils, plants and fruits with anti-inflammatory effects and expected anti-SARS-CoV-2 effects from their binding affinity to SARS-CoV-1 or 2, or from results of in vitro assays.

Oils, Plants, Fruits Type	Major Chemical Constituent	Confirmed Effects and Binding Affinities
Ashitaba (*Angelica keiskei*)	**4-hydroxyderricin** (CID: 6438503; CAS: 55912-03-3)	**Anti-viral**: [411]
	**Xanthoangelol** (CID: 643007; CAS: 62949-76-2)	**Anti-viral**: [411,425]
Bitter orange (*Citrus aurantium* L. (Rutaceae))	**Limonene**	**Anti-inflammation** [275,278,297]
	**Linalool**	**Anti-inflammation** [19,20,21,22,275,278,290,296,297]
	***β*-Myrcene**	**Anti-inflammation** [275,278,337]
	**Kaempferol**	**Anti-inflammation** [315]
	**Quercetin**	**Anti-viral**: [411,423]
	**Luteolin**	**Anti-viral**: COV2 by binding affinity to 3CL^pro^ [421]; to 3pro and ACE2 [422]
Cinnamon (*Cinnamomum*)	**Camphor**: 60% in root bark [426]	**Anti-inflammation** [278,296]
	**Cinnamaldehyde**: 65–80% in bark [426]	**Anti-viral**: [408]
	***Z*-Cinnamyl acetate**: 42–54% in fruit [426]; 11.85% in *Cinnamomum verum* oil [427]	**Anti-viral**: [408]
	***E*-Cinnamyl acetate**: 41.98% in flowers of Ceylon cinnamon, *Cinnamomum zeylanicum* [426]; 11.78% in *Cinnamomum verum* oil [427]	**Anti-viral**: [408]
	**Eugenol**: 70~95% in leaves [428], *Cinnamomum zeylanicum* [426]	**Anti-inflammation** [296,346,347,348]
	**Linalool**: 16.85% in *Cinnamomum verum* oil [427]	**Anti-inflammation** [19,20,21,22,275,278,290,296,297]
Citronella oil	See lemongrass	
Clove (*Syzygium*)	**ß-Caryophyllene**: 17.4% in leaf [429], 1.39% in bud [430], 14.84% and 12.79% in bud from Java and Manado, Indonesia, respectively [431]; 4.5% in oil [432]	**Anti-inflammation** [16,278,319]
	**Eugenol**: 88.6% in bud [430], 74.65% and 55.65% in bud from Java and Manado, Indonesia, respectively [431], 76.8% in leaf [429], 69.4% in bud [433]; 85.7% in oil [432]	**Anti-inflammation** [296,346,347,348]
	**Eugenyl acetate**: 10.79% [433], 1.2% in leaf [429], 20.54% and 8.7% in bud from Java and Manado, Indonesia, respectively [431]	**Anti-inflammation** [348]
	**α-Humulene**: 2.1% in leaf [429], 2.75% and 1.53% in bud from Java and Manado, Indonesia, respectively [431]	**Anti-inflammation** [276,278,360]
Copaiba (*Copaifera*)	**ß-Caryophyllene**: 24.9% in oil [434], 21.7% in oil [435]	**Anti-inflammation** [16,278,319]
	**α-Bergamotene**: 20.5% in oil [435]	-
	**ß-Bisabolene**: 23.6% in oil [435]	-
	**α-Humulene**: 2.9% in oil [435]	**Anti-inflammation** [276,278,360]
	**Caryophyllene oxide**: 4.1% in oil [435]	**Allergen**
Cypress (Hinoki) (*Chamaecyparis obtusa*)	**α-cadinol**: 10.9% in oil [436]	**Anti-viral** [407]
	**Borneol**: 16.0% in oil [436]	**Anti-inflammation** [275,278,319]
	**Dehydroabieta-7-one**	**Anti-viral** [407]
	**6,7-Dehydroroyleanone**	**Anti-viral** [407]
	**Ferruginol**: inclusion and % varies largely	**Anti-inflammation** [309]
	**ß-Hydroxyabieta-9(11)**	**Anti-viral** [407]
	**α-Terpineol**: 19.4% in oil [436]	**Anti-inflammation** [275,278,319,387,388]
Elderberry (*Sambucus nigra* L.)	**Quercetin**	**Anti-viral** [411,423]
	**Rutin**	**Anti-viral** [412,423]
	**Kaempferol**	**Anti-inflammation** [437]
	**Caffeic acid**	**Anti-inflammation** [437]
	**3,4-dihydroxyphenylacetic acid**	**Anti-inflammation** [437]
Eucalyptus (*Eucalyptus*)	**Borneol**: 5.5% in leaf and 5.5% in fruit [438]	**Anti-inflammation** [275,278,319]
	**1,8-Cineole (eucalyptol)**: large percentage difference among species and countries of origin, 2.9% to 90.0% [439], species percentage differences in leaves from 49.07 to 83.59% [300], 14.1% in leaf and 34.5% in fruit [438]	**Anti-inflammation** [11,238,241,261,262,265,267,291,400]
	***p*-Cymene**: 42.1% in leaf and 30.0% in fruit [438]	**Anti-inflammation** [322,328]
	**Limonene**: 5.5% in leaf and none in fruit [438], 1.33% in *E. lehmani* leaves and 3.32% in *E. sideroxylon* leaves [300], from 0% to 28% depending on the species and location [439]	**Anti-inflammation** [275,278,297]
	**α-Pheliandrene**: from 0% to 20.1% depending on the species and location [439]	**Anti-inflammation** [278]
	**α-Pinene**: 12.7% in leaf and 9.0% in fruit [438], from 1.27% to 26.35% in 7 Eucalyptus species’ leaves with highest in *E. lehmani* [300], from 0% to 52.7% depending on the species and location [439]	**Anti-inflammation** [278,337]
	**Spathulenol**: 3.2% in leaf and none in fruit [438], 1.15% in *E. astrengens* leaves [300], from 0% to 41.5% depending on the species and location [439]	**Anti-inflammation** [387]
	**γ-Terpinene**: none in leaf and 5.1% in fruit [438], from 0% to 29.2% depending on the species and location [439]	**Anti-inflammation** [278,360,386]
Geranium (*Pelargonium*)	**ß-bourbonene**: 2.7% in oil from Tajikistan [440]	-
	**Caryophyllene oxide**: 3.7% in oil from Tajikistan [440]	**Allergen** [441]
	**Citronellol**: 37.5% in oil from Tajikistan [440]	**Anti-inflammation** [275,278,290,291] **Anti-viral**: Down-regulated expression of ACE2 and TMPRSS2,
	**Geraniol**: 6.0% in oil from Tajikistan [440]	**Anti-inflammation** [275,278,290,291] **Anti-viral**: [408]
	**Geranyl formate**: 2.0% in oil from Tajikistan [440]	
	**Linalool**: 3.0% in oil from Tajikistan [440]	**Anti-inflammation** [19,20,21,22,275,278,290,296,297]
Ginger (*Zingiber officinale* Roscoe)	**6-Gingerol**: (23–25%) [442] Other chemical constituents: **10-gengerol, 6-shogaol, α-Terpinene, α-Terpineol, 4-Terpineol, Terpinolene, γ-Terpinolene, Cineole, Nerol, Borneol, Citronellol, Geraniol, Linalool, Camphor, Neral**	**Anti-viral**: [409]
Lavender (*Lavandula*)	**Borneol**: 0.3% to 22.4% [443]	**Anti-inflammation** [275,278,319]
	**Camphor**: none to 11.76% [443]	**Anti-inflammation** [278,296]
	**ß-Caryophyllene**: none to 3.2% [443]	**Anti-inflammation** [16,278,319]
	**1,8-Cineole (Eucalyptol)**: 0.1% to 10.89% depending on location [443]	**Anti-inflammation** [275,278,296,297,300,302,303]
	**Geraniol**: none to 11.02% [443]	**Anti-inflammation** [275,278,290,291] **Anti-viral**: [408]
	**Lavandulol**: 3.7% in oil [444]	-
	**Lavandulol acetate** (lavandulyl acetate): 0.2% to 21.6% in oils depending on location [445]; 5.7% in oil [444]; 12.68% in oil [446], none to 10.78% [443]	-
	**Linalool**: 49.9% in oil [444]; 19.71% in oil [446], 4.91% to 57.48% [443]	**Anti-inflammation** [19,21,22,275,278,290,296,297]
	**Linalyl acetate**: 9.3% to 68.8% in oils depending on location [445]; 14.4% in oil [444]; 26.61% in oil [446], none to 35.39% [443]	**Anti-inflammation** [275,297,364]
	**Myrcene**: 1.4% in oil [444]	**Anti-inflammation** [275,278,337]
	**Ocimene**: 2.4% to 2.6% in oil [444]	**Anti-inflammation** [17,337,372]
	**4-Terpineol, terpinen-4-ol (isomer of terpineol)**: 0.1% to 5.8% in oils depending on location [445], none to 8.07% [443]	**Anti-inflammation** [275,278] **Anti-viral**: [408]
Lemongrass (*Cymbopogon citratus*)	**ß-Caryophyllene**: 3.26% in oil [447], 1.09 in oil [338]	**Anti-inflammation** [16,278,319]
	**Citral A** (**Geranial**, *E*-isomer of citral): 37.40% in oil from Guangxi, China [448], 26.1% in oil [447], 37.58 to 45.95% in oil [449], 40.16% in oil [338]	**Anti-inflammation** [275,278,284,290,337]
	**Citral B** (**Neral**, *Z*-isomer of citral): 31.97% in oil from Guangxi, China [448], 31.5% in oil [447], 29.44 to 31.13% in oil [449], 34.24% in oil [338]	**Anti-inflammation**: [336]
	**Citronellol**: 1.10% in oil from Guangxi, China [448], 2.95% in oil [447], 0.35% to 0.51% in oil [449]	**Anti-inflammation** [275,278,290,291]
	**Geraniol**: 1.55% in oil from Guangxi, China [448], 2.15% in oil [447], 5.11% in oil [338]	**Anti-inflammation** [275,278,290,291] **Anti-viral**: [408]
	**Geranyl acetate**: 1.06% to 2.16% in oil [449], 2.27% in oil [447], 2.89% in oil [338]	**Anti-inflammation** [297,332]
	**Juniper camphor**: 1.28 to 2.82% [449]	-
	**Limonene**: 0.65% in oil from Guangxi, China [448], 2.32% in oil [447], 0.33% in oil [338]	**Anti-inflammation** [275,278,297]
	**Linalool**: 1.12% in oil from Guangxi, China [448], 0.58% to 0.87% [449], 1.45% in oil [338]	**Anti-inflammation** [19,20,21,22,275,278,290,296,297].
	**Myrcene**: 15.65% in oil from Guangxi, China [448]	**Anti-inflammation** [275,278,337]
Licorice (*Glycyrrhiza* spp.)	Over 20 triterpenes and 300 flavonoids of natural active compounds and 73 bioactive compounds identified. “3 triterpenes, 18b-GC, 18a-GC and 18b-glycyrrhetinic acid (18b-GA), and 13 flavonoids, licochalcone A (LCA), licochalcone B (LCB), licochalcone C (LCC), licochalcone D (LCD), licochal- cone E (LCE), isoliquiritigenin (ISL), echinatin (EC), glabridin (GLD), isoangustone A (ISOA), licoricidin (LID), licorisoflavan A (LIA), dehydroglyasperin C (DGC) as well as dehydroglyas- perin D (DGD), all have been reported to possess anti-inflammatory activity.” [356]; contained in root twigs	**Anti-inflammation** [356,357] **Anti-viral**: [417,418]
Mint, mentha (spearmint, *Mentha spicata*)	**Carvone**: 40.8% in oil [450], 49.5% [451], 70.36% in oil [452]	**Anti-inflammation** [275,297,328,329]
	**ß-Caryophyllene**: 1.2% in oil [450], 2.7% in oil [451], 1.1%. in oil [452]	**Anti-inflammation** [16,278,319]
	**1,8-Cineole** (**Eucalyptol**): 17.0% in oil [450], 8.7% in oil [451], 2.24% in oil [452]	**Anti-inflammation** [275,278,296,297,300,301,302,303,332]
	**Limonene**: 20.8% in oil [450], 16.1% in oil [451], 6.6% in oil [452]	**Anti-inflammation** [275,278,297]
	**ß-Pinene**: 2.2% in oil [450], 1.1% in oil [451], 0.6% in oil [452]	-
	**4-Terpineol**: 1.3% in oil [450], 1.5% in oil [451], 1.09% in oil [452]	**Anti-inflammation** [275,278] **Anti-viral**: [408]
Oregano (*Poliomintha longiflora*)	**Carvacrol**: 12.6% in oil, 60.03% to 64.315 in fractions at 140C and undistilled oil, respectively [453]	**Anti-inflammation** [275,285,326,337] **Anti-viral**: [408]
	***P*-Cymene**: 11.5% to 35.7% in *Oreganum vulgare* in Nefza, Tunisia and different harvest years, 27.3% to 46.3% in Krib, Tunisia and different harvest years [454,455]	**Anti-inflammation** [322,328]
	**o-Ocymene**: 39.13% in oil, 47.96 to 53.97% in fractions at 82C and 100C, respectively [453]	-
	**α-Terpinene**: 5.57% in oil [453]	**Anti-inflammation** [275,360]
	**γ-Terpinene**: 22.34% in oil, 15.59%, 24.43%, 40.57% in fractions at 82C, 100C, 120C, respectively [453]	**Anti-inflammation** [278,360,386]
	**Thymol**: 1.71% in oil, 5.08% and 3.77% in fractions at 140C and undistilled oil, respectively [453]	**Anti-inflammation** [275,319,337,389] **Anti-viral** [408]
Paper mulberry (*Broussonetia papyrifera*)	**Bavachromene** (CID: 5321800)	**Anti-viral**: [411]
	**3′-(3-methylbut-2-enyl)-3′,4,7-trihydroxyflavane** (CID: 129704069)	**Anti-viral**: [411]
	**Broussoflavan A** (CID: 44257078)	**Anti-viral**: [411]
	**Kazinol A** (CID: 442414; CAS: 99624-28-9)	**Anti-viral**: [411]
	**Kazinol B** (CID: 480869; CAS: 99624-27-8)	**Anti-viral**: [411]
	**Kazinol J** (CID: 21637732)	**Anti-inflammation**: [320] **Anti-viral**: [420]
	**Broussonol E** (CID: 10343070)	**Anti-viral**: [411]
	**Broussoflavonol B** (CID: 480828)	**Anti-inflammation**: [320]
	**Others**: polyphenols (broussochalcone A, papyriflavonol A, 3′-(3-methylbut-2-enyl)-3′,4′,7-trihydroxyflavane, kazinol F	**Anti-inflammation**: [456,457] **Anti-viral**: [420]
Peppermint (hybrid mint, *Mentha × piperita*, or *Mentha balsamea*)	**ß-Caryophyllene**: 1.7% in oil [458]	**Anti-inflammation** [16,278,319]
	**1,8-Cineole**: 5.3% in oil [458]; 5.13% in oil [459]; 5.62% in oil [452]	**Anti-inflammation** [275,278,296,297,300,301,302,303,332]
	**Limonene**: 2.6% in oil [458]; 1.58% in oil [452]	**Anti-inflammation** [275,278,297]
	**Menthol**: 40% in oil [458]; 36.02% in oil [459]; 38.45% in oil [452]	**Anti-inflammation** [275,300,337,366,367] **Anti-viral**: [408]
	**Menthone**: 23.4% in oil [458]; 24.56% in oil [459]; 21.85% in oil [452]	**Anti-inflammation** [275,368]
Rosemary (*Rosmarinus officinalis*)	**Borneol**: 3% in oil [432]; 4.08% to 8.17% depending on the location in Tunisia [460]	**Anti-inflammation** [275,278,319]
	**ß-Caryophyllene**: 3.2% in oil [432]	**Anti-inflammation** [16,278,319]
	**1,8-Cineole (Eucalyptol)**: 43.1% in oil [432]; 33.08% to 37.75% depending on the location in Tunisia [460]	**Anti-inflammation** [275,278,296,297,300,301,302,303,332]
	**Camphor**: 11.3% in oil [432]; 13.55% to 18.13% depending on the location in Tunisia [460]	**Anti-inflammation** [278,296]
	**α-Pinene**: 11.4% in oil [432]; 8.58% to 9.32% depending on the location in Tunisia [460]	**Anti-inflammation** [278,337]
	**Camphene**: 5.0% in oil [432]; 3.58% to 5.07% depending on the location in Tunisia [460]	**Anti-inflammation** [322,328]
	**Limonene**: 2.6% in oil [432]; 2.99% to 3.19% depending on the location in Tunisia [460]	**Anti-inflammation** [275,278,297]
Summer savory (*Satureja hortensis*)	**Carvacrol**: 11% to 67.0% depending on the location harvested [461]; 2.5% in oil [462]	**Anti-inflammation** [275,285,326,337] **Anti-viral**: [408]
	***P*-Cymene**: 3.4% to 11.7% in oil depending on the location harvested [461]; 6.30% [462]	**Anti-inflammation** [322,328]
	**Γ-Terpinene**: 15.30% to 38.7% depending on the location harvested [461]; 20.72% [462]	**Anti-inflammation** [278,360,386]
	**ß-myrcene**: 1.9% to 2.8% depending on the location harvested [461]; 1.98% [462]	**Anti-inflammation** [275,278,337]
	**α-Terpinene**: 1.29% to 4.9% depending on the location harvested [461]; 2.93% [462]	**Anti-inflammation** [275,360]
	**4-Terpineol**: none to 1.6% depending on the location harvested [461]; 0.17% [462]	**Anti-inflammation** [275,278] **Anti-viral**: [408]
	**Thymol**: 23.12% [462]; none to 28.2% [461]	**Anti-inflammation** [275,319,337,389] **Anti-viral**: [408]
Tea tree or Narrow-leaved paperbark (*Melaleuca alternifolia*)	**ß-Caryophyllene**: 2.2% in oil [432]	**Anti-inflammation** [16,278,319]
	***p*-Cymene**: 2.8% in oil [432]; 2.9% in oil [463]	**Anti-inflammation** [322,328]
	1,8-Cineole (eucalyptol): 2.3% in oil [432]; 5.1% in oil [463]	**Anti-inflammation** [275,278,296,297,300,301,302,303,332]
	**Limonene**: 3.6% in oil [432]; 1.0% in oil [463]	**Anti-inflammation** [275,278,297]
	**α-Pinene**: 3.9% in oil [432]	**Anti-inflammation** [278,337]
	**4-Terpineol**: 38.7% in oil [432]; 40.1% in oil [463]	**Anti-inflammation** [275,278] **Anti-viral**: [408]
	**Γ-Terpenine**: 16.3% in oil [432]; 23.0% in oil [463]	**Anti-inflammation** [241,318,344]
	**α-Terpineol**: 4.6% in oil [432]; 2.4% in oil [463]	**Anti-inflammation** [275,278,319,387]
Tea plant (*Camellia sinensis*)	**(+)-Catechin**	**Anti-inflammation** [330]
	**(−)-Epicatechin**	**Anti-viral**: [413]
	**Epigallocatechin-3-gallate**	**Anti-inflammation** [345] **Anti-viral**: [416]
Thyme (*Thymus vulgaris*)	**α-Phellandrene**: 0.33% [464]; 0.3% [465]; 0.27% [466]	**Anti-inflammation** [278]
	**α-Pinene**: 1.07% [464]; 0.8% [465]; 0.47% [466]; 1.6% in oil [432]	**Anti-inflammation** [278,337]
	**α-Terpinene**: 2.79% [466]	**Anti-inflammation** [275,360]
	**γ-Terpinene**: 30.9% [464]; 16.5% [465]; 29.12% [466]; 7.9% in oil [432]	**Anti-inflammation** [278,360,386]
	**Thymol**: 47.59% [464]; 44.7% [465]; 43.1% in oil [432]	**Anti-inflammation** [275,319,337,389] **Anti-viral**: [408]
Turmeric (*Curcuma longa*)	**Curcumin**: Curcumin vs. demethoxycurcumin vs. bisdemothoxycurcumin is 80:15:5 but some reports suggest demethoxycurcumin has stronger bioactive potency [339]	**Anti-inflammation**: [339,340,341] **Anti-viral**: [341,412,413,467]
	**demethoxycurcumin**	-
	**Bisdemothoxycurcumin**	-
	**Cyclocurcumin**	**Anti-inflammation**: [341,342] **Anti-viral**: [341]

-: none or not so many studies, or no in vitro and/or in vivo studies at chemical compound level to be convincing.

### 7.5. Phytochemicals for Neuropharmacological Effects

Patients who experience anosmia/hyposmia often experience anxiety and depression as well. Often this is considered to be caused by worrying about if they will recover and because of the acute stress and confusion due to the sudden loss of the senses of smell and taste. There are, however, other possible reasons that can cause anxiety and depression after losing the senses, i.e., the lack of neuroendocrinological modulation by odors, which usually takes place and affects/regulates our physiological conditions in normosmia conditions.

A well-known example on the influence of body odors is their influence on the menstrual cycles of women, which was first reported in the 1970s [468]. Later studies found that odors of women in the late follicular phase accelerated the secretion of luteinizing hormone in recipient women [469]. Odors of women in the ovulatory phase also had influences on the secretion of testosterone in men [470,471], showing that the physiological conditions of both sexes are under the control of odors of others. Studies on behaviors [472] and the brain [473,474] have shown that influences of odors can happen at even subliminal levels [473] and in a reciprocal way [474]. Men exposed to human tears from women showed reduced levels of testosterone and less sexual arousal when they were exposed to pictures of women, although the tears were perceived odorless [475]. Studies have also shown that underarm odors of men stimulate the secretion of luteinizing hormone in women and increase the feeling of relaxation [476], indicating that odors affect mood. Women who experience unexplained repeated pregnancy loss had an altered olfactory sense and olfactory responses to men’s body odors [477], suggesting that olfactory sense affects the process of pregnancy as well. Studies on embryos and neonates have shown that odors play significant roles from early stages in life to adolescence affecting our physiological condition, our emotions, and behaviors [478]. These studies suggest that losing the sense of smell is not only a situation where patients are distressed by losing their senses or by being unable to enjoy the smell of food, but it is a situation in which the patients are deprived of the effects of odors on the physiological conditions that are produced in the normosmia condition. The anxiety and depression could be caused by multiple reasons, including the changes caused by the lack of the olfactory regulation of the physiological conditions, other than the distress from the sudden loss of the senses, and the frustration by being unable to smell foods and other odors.

Phytochemicals, other than body odors, have also been known for centuries to have effects on moods and physiological conditions, although scientific studies on these influences started only recently. There are studies suggesting that multiple routes are possibly involved to cause neuroendocrinological changes, resulting in changes in moods and physiological conditions: the olfactory system, the trigeminal neurons, and through absorption to the skin and digestive system. For example, exposure to the odor of linalool, which is one of the major chemical components of lavender essential oil, induced significant analgesic [479] and anxiolytic [19] effects in mice, and these effects diminished in the mice deprived of olfactory input. Although the linalool receptors responsible for these analgesic and anxiolytic effects are not identified yet, involvement of the olfactory system was suggested from the expression of an immediate early gene (c-Fos, Arc, phosphorylated ERK) in the granule cells and periglomerular cells of the main olfactory bulb in mice after exposure to linalool. There are trigeminal nerves in the nasal cavity and some odorous molecules inhaled into the nostril activate the trigeminal nerves [480]. Although there are studies reporting that several types of TRP channels (TRPM8, TRPA1) expressed on the trigeminal nerves are activated by linalool [20,22,481], there are also studies suggesting that the linalool odor-induced analgesia was triggered by a TRPA1-independent pathway [482]. As another pathway, odorous compounds absorbed via mucosal membranes can reach the CNS and directly cause neuropharmacological effects. In the case of linalool, after an hour of inhalation of vaporized linalool, the serum concentration of linalool reached 4.22 ng/mL in mice [483]. Part of such absorbed compounds reached the brain, crossing the blood-brain barrier. A number of studies showed that systemic administration of linalool induced analgesic, anxiolytic, and other physiological effects [484]. Though it is still under debate whether the serum concentration of linalool could reach the effective dose (cf, 50–200 mg/kg for analgesia) by linalool inhalation, it is possible that the absorbed compounds cause neuropharmacological effects in the CNS.

Studies using human subjects have shown comparative results [485]. Adult human subjects who inhaled (R)(−)-linalool showed reduced heart rate and produced a calm and vigorous mood [485]. Exposure to lavender, which contains linalool, reduced stress levels and anxiety, enhanced positive moods, increased a relaxing mood, and increased the percentage of deep slow wave sleep [486].

These studies suggest that if there are multiple routes involved in the effects of phytochemicals, smell training may have positive influences on anxiety and depression as well and this needs to be addressed by future studies. Anxiety and depression, which are often observed following the loss of senses, could have multiple causes, including the possibility the loss of the sense caused a secondary impact of the loss of regulation of physiological conditions through odors. This is also one of the possible issues that needs to be addressed in future studies. There are few studies on the influences of the loss of the senses of smell and taste on physiological conditions. Following the large increase in the number of anosmia and ageusia patients caused by COVID-19, there is a strong need for studies in this unexplored area.

## 8. General Discussions

In this review, we have summarized what is known of the olfactory sense, taste sense, COVID-19-induced anosmia and ageusia, smell training using essential oils, and the phytochemicals that can be used in selecting the essential oils and diets for smell training and taste training from their bioactive properties. We believe this is the first review in which the bioactive properties and phytochemicals in the essential oils as well as diets are discussed from the viewpoint of facilitating recovery from anosmia/hyposmia and ageusia/hypogeusia. Based on the summary of the chemical compounds we have reviewed, we would like to propose a new combination of essential oils for smell training for COVID-19-induced anosmia/hyposmia and a new taste training for COVID-19-induced ageusia/hypogeusia.

### 8.1. A New Smell Training Essential Oil Combination for COVID-19-Induced Anosmia/Dysnomia 

The traditional combination of essential oils for smell training has been rose (flowery), lemon (fruity), cloves (aromatic), and eucalyptus (resinous) [13]. Table 5 is a simplified summary of the essential oils with chemical constituents with expected anti-CoV1 or 2 effects as well as those with anti-inflammatory effects based on Table 4. There are perhaps more included in these herbs and plants, and the numbers in Table 4 indicate the numbers covered in this review. There will also be seasonal differences, geographic differences, and differences depending on the parts of the plants in the chemical constituents [17]. When oils and diets are selected, it is necessary to confirm the chemical constituents because of these reasons and these differences can cause differences in their effects.

If we select the essential oils with more than 5 anti-inflammatory chemical constituents and at least 1 anti-viral chemical constituent, there are 11 types of essential oils, which are paper mulberry, licorice, turmeric, lavender, thyme, summer savory, lemongrass, tea tree, oregano, mint, and peppermint. These are the candidate essential oils with higher numbers of chemical constituents with anti-inflammatory and anti-viral effects. Paper mulberry, hinoki cypress, summer savory, and cinnamon have the highest number of anti-viral chemical constituents. The herbs with anti-viral effects but containing a rather lower number of chemical constituents with anti-inflammatory effects could be effective to use in combination with other essential oils which contain many different types of chemical constituents with anti-inflammatory effects, for example, combining them with lavender, lemongrass, thyme, tea tree, or eucalyptus. If we consider combining these results on the bioactive properties and the classic four types of smell classification of flowery, fruity, aromatic, and resinous, the combinations of four types of essential oils could be, for example, lavender (flowery), lemongrass (fruity), hinoki cypress (woody/resinous).

**Table 5:** Summary of the number of chemical constituents with anti-viral and anti-inflammatory effects listed in this study.

Essential Oil/Herbs/Plants/Fruits	Anti-Viral for SARS-CoV-1 or 2	Anti-Inflammation	Comments
Ashitaba	2	-	
Bitter orange	2	4	
Cinnamon	3	3	
Clove	0	4	
Copaiba	0	2	Contains ß-caryophyllene, which is a CB2 ligand, at high %. Possibly good especially for tongue regeneration from the genes involved in turnover in the epidermis of skin and the pathways that ß-caryophyllene activates.
Cypress (hinoki)	4	3	
Elderberry	2	3	
Eucalyptus	0	8	
Geranium	2	3	
Ginger (fresh)	1	at least 2	
Lavender	2	10	
Lemongrass	1	9	
Licorice	2	Many	
Mint	1	5	
Oregano	2	5	
Paper mulberry	Many	Many	
Peppermint	1	5	
Rosemary	0	7	
Summer savory	3	7	
Tea tree	1	8	
Tea plant	2	1	
Thyme	2	9	
Turmeric	2	Many	

### 8.2. A New Taste Training for COVID-19-Induced Ageusia/Dysgeusia 

The choices of phytochemicals become more numerous for taste training because it is not necessary to limit the choices to volatile chemical compounds. Concentration (dilution rate) and sterilization become important as they will be consumed and also because they will be placed directly in the mouth. Possible considerations in developing a new taste training combination could be (1) add chemical constituents with anti-inflammatory effects and anti-coronavirus effects in addition to (2) basic types of taste, and (3) a structure that can stay in the mouth for a rather long time, for example, candies and/or chewing gums, to lengthen the time that the gustatory system is exposed to the chemical constituents. For example, candies or chewing gum with sweet, sour, salty, or bitter taste added with chemical constituents with anti-inflammatory and anti-coronavirus effects, and, for example jerkies for training of umami taste with chemical constituents containing anti-inflammatory and anti-coronavirus effects.

### 8.3. Possible Utilization of Phytochemicals as Supplementary Treatments in Clinical Settings

A variety of drugs such as zinc sulfate, Chinese medicines, oral and intranasal steroids, and vitamins have been used for the treatment of PVOD. Furthermore, α-lipoic acid, minocyclin and theophylline were tested in clinical trials [161]. However, none of these medicines have been proven as truly effective by studies with more stringent designs, such as double-blind, randomized, placebo-controlled trials. Smell training is, therefore, already an important therapeutic component in the clinical management of PVOD. It is also recommended by experts in olfactory medicine as a promising therapeutic method for COVID-19 anosmia [273,487].

Although the pathophysiology of PVOD in humans and COVID-19-induced anosmia remains largely unclear, research on experimental animals suggest that inflammatory tissue damage in the olfactory neuroepithelium and secondary changes in the central olfactory pathway could be involved in the pathogenesis. Therefore, if we consider the biological effects of essential oils along with the effects of olfactory stimulation, such as anti-inflammatory effects, activation of cell proliferation, and promotion of nerve axon extension, we may be able to establish a more effective smell training.

As for post-viral gustatory dysfunction, there is no taste training method that corresponds to smell training. It is necessary to conduct both basic research on gustatory dysfunction in order to determine the causations and clinical application of the candidate taste training methods for the development of taste training. The anti-inflammatory and anti-CoV-2 effects of phytochemicals may contribute to facilitating recovery.

### 8.4. Possible Utilization of Phytochemicals for Prevention of Contracting SARS-CoV-2 and Treatment of COVID-19

Opportunities to utilize phytochemicals are not limited to smell/taste training or as supplemental treatments in clinical settings. By modifying the concentrations and chemical stability, it might be possible to develop various agents which will help prevent contraction of the virus, such as a coating on masks, a spray for surfaces, diffusers to reduce virus in airborne particles, and so on.

The lists of phytochemicals show the candidates and thorough tests on their efficacy are required before utilizing them in the prevention and treatment for COVID-19. This is because some of the chemical compounds with high binding affinity do not have sufficient anti-viral effects. A good example is hydroxychloroquine. Hydroxychloroquine was considered to have high anti-viral effects due to its binding affinity, and it turned out to be not as effective as expected. The variants also may have different binding affinities as well. In an emergency situation, such as a pandemic, a possible way to accelerate their use would be to start using phytochemicals with no side effects, that are Food Grade nutrients, and used in combinations instead of as a single additive in order to enhance the chances that it works, and meanwhile proceed on testing other candidates.

### 8.5. Allergen Cautions 

Chemical compounds that cause no or few allergic reactions may get oxidized and the oxidized compound may cause an allergy. An example is ß-caryophyllene. ß-caryophyllene is known to cause no or a minimum level of allergic responses, but the oxidized chemical compound caryophyllene oxide is well known to cause allergic responses at moderate strength [441]. Oxidation of ß-caryophyllene was found to start immediately after exposure to air and reduced to 50% in 5 weeks [441]. Citronellol is also known to oxidize and increase the possibility of sensitization, most likely by citronellol hydroperoxides [488]. Geraniol, R-limonene, and linalool are also known to have sensitizing capacities [441,489,490]. These studies indicate the importance of considering oxidization and sensitization, the stability of the chemical compounds, and the method to enhance stability for each of the chemical compounds to be used. One of the possible ways to enhance stability is, e.g., utilization of cyclodextrin and establish an inclusion complex, although this can reduce the bioactive property. Measurement of the binding strength of the host and guest chemical compounds and obtaining the time course for release for each host-guest combination will provide information which will help in choosing the host, for example, the type of cyclodextrin.

## 9. Concluding Remarks

In this review, we have summarized the morphology and physiology of the olfactory and gustatory system, COVID-19-induced anosmia and ageusia, and the phytochemicals that have anti-inflammatory and anti-viral effects, which may facilitate recovery from COVID-19-induced loss of senses. Bioactive properties of essential oils used in smell training have been completely overlooked so far. Although it is hard to go through all the phytochemicals and all the herbs, spices, and other medicinal plants that contain phytochemicals, we hope that this review can provide a meaningful start in reconsidering the roles of essential oils in smell training from the perspective of the bioactive properties of their chemical constituents. Inflammation is one of the major symptoms of COVID-19. The anti-inflammatory effects of various phytochemicals suggest a promising possibility in facilitating recoveries from COVID-19-induced anosmia and ageusia. There are reports already showing that smell training using essential oils improves recovery from anosmia even though the chemical constituents were not considered in the selection of the oils. Essential oils, including the oils used in smell training (rose, lemon, clove, eucalyptus), contain several hundred chemical constituents [17]. If the bioactive properties in these oils are involved in such improvements, selection of oils based on the bioactive properties of the chemical constituents may produce better improvements. This needs to be determined in future studies in experimental settings. The same effects can be expected in facilitating recovery from ageusia and the agents do not need to be essential oils. They can be diets and drinks. We are still at the starting point for studies on taste training and what to use for them, which also requires input from the studies on causations. We hope that this study will be a road sign to guide much needed studies with a perspective on the bioactive properties of phytochemicals.

## Data Availability

Not applicable.
